# Single-nuclei transcriptome analysis of IgM^+^ cells isolated from channel catfish (*Ictalurus punctatus*) spleen

**DOI:** 10.3389/fimmu.2025.1547193

**Published:** 2025-03-17

**Authors:** Johanna E. Aldersey, Jason W. Abernathy, Benjamin H. Beck, Miles D. Lange

**Affiliations:** ^1^ ARS Research Participation Program, Oak Ridge Institute for Science and Education (ORISE), Oak Ridge, TN, United States; ^2^ Aquatic Animal Health Research Unit, Agricultural Research Service (ARS), United States Department of Agriculture, Auburn, AL, United States

**Keywords:** single-cell RNA sequencing, single-nuclei RNA sequencing, B cell, plasma cell, T cell, lymphocyte, natural killer-like, hematopoietic stem and progenitor cells

## Abstract

Catfish production is the primary aquaculture sector in the United States, and the key cultured species is channel catfish (*Ictalurus punctatus*). The major causes of production losses are pathogenic diseases, and the spleen, an important site of adaptive immunity, is implicated in these diseases. To examine the channel catfish immune system, single-nuclei transcriptomes of sorted and captured IgM^+^ cells were produced from adult channel catfish. Three channel catfish (~1 kg) were euthanized, the spleen dissected, and the tissue dissociated. The lymphocytes were isolated using a Ficoll gradient and IgM^+^ cells were then sorted with flow cytometry. The IgM^+^ cells were lysed and single-nuclei libraries generated using a Chromium Next GEM Single Cell 3’ GEM Kit and the Chromium X Instrument (10x Genomics) and sequenced with the Illumina NovaSeq X Plus sequencer. The reads were aligned to the *I. punctatus* reference assembly (Coco_2.0) using Cell Ranger, and normalization, cluster analysis, and differential gene expression analysis were carried out with Seurat. Across the three samples, approximately 753.5 million reads were generated for 18,686 cells. After filtering, 10,637 cells remained for the cluster analysis. The cluster analysis identified 16 clusters which were classified as B cells (10,276), natural killer-like (NK-like) cells (178), T cells or natural killer cells (45), hematopoietic stem and progenitor cells (HSPC)/megakaryocytes (MK) (66), myeloid/epithelial cells (40), and plasma cells (32). The B cell clusters were further defined as different populations of mature B cells, cycling B cells, and plasma cells. The plasma cells highly expressed *ighm* and we demonstrated that the secreted form of the transcript was largely being expressed by these cells. This atlas provides insight into the gene expression of IgM^+^ immune cells in channel catfish. The atlas is publicly available and could be used garner more important information regarding the gene expression of splenic immune cells.

## 1 Introduction

In modern agriculture, there is a drive to improve the sustainability of production systems due to population growth and limitations in resources (1, 2). Catfish production is the primary aquaculture sector in the United States and in 2023 the industry generated $437 million in sales (3). The two main cultured species are channel catfish (*Ictalurus punctatus*) and channel ♀ x blue ♂ catfish (*Ictalurus furcatus*) hybrids. The primary cause of production losses are pathogenic diseases, particularly the bacterial diseases motile *Aeromonas* septicemia, columnaris disease, and enteric septicemia (4, 5). These diseases impact the sustainability and efficiency of the catfish production systems and producers require new disease control methods and interventions. In addition to industry significance, channel catfish are also an important comparative model of the teleost immune system (6–10). Therefore, there is interest in a further understanding of the catfish immune system.

The primary systemic organs of the teleost immune system such as channel catfish are the head kidney, thymus, and spleen (11). The head kidney (also known as the anterior kidney) has a function equivalent to mammalian bone marrow (11). It maintains stem cells that differentiate into erythrocytes and immune cells (11). The thymus, similarly to the mammalian thymus, trains and matures T cells (11). The spleen is a secondary lymphoid tissue, and the main function of the organ is to provide an environment for lymphocytes, B cells and T cells, and other immune cells; filter the blood of antigens and pathogens; and maintain erythrocyte homeostasis (12). B cells are adaptive immune agents that detect antigens through the B cell receptor (BCR) (13). Antigen binding, along with a “second signal”, most notably from T helper cells, activates B cells to differentiate into antibody secreting cells, plasmablasts, and plasma cells (13, 14).

The spleen and B cells are implicated in two of the key pathogenic diseases of catfish. The channel catfish spleen, after exposure in challenge trials, harbor virulent *Aeromonas hydrophila* (vAh) (15), the pathogen that causes motile *Aeromonas* septicemia, and *Edwardsiella ictaluri* (16), the pathogen that causes enteric septicemia of catfish (ESC). In addition, yellow catfish (*Pelteobagrus fulvidraco*) hybrid challenged with *A. hydrophila* with vAh had altered B cell gene expression in the head kidney when compared to the control (17).

Single-cell/single-nuclei RNAseq (sc/snRNAseq) enables the characterization of gene expression at the individual cell level, which is highly desirable for the research of homogenous tissues. This contrasts with bulk RNAseq, where the transcriptome averages the gene expression across multiple cells and cell types. This means that rare cell types and lowly expressed genes are often masked within the data (18). sc/snRNAseq enables these rare cell types to be discovered and individual cells to be profiled based on their gene expression. Previously, our group developed a single nuclei transcriptomic atlas of the *I. punctatus* spleen (19). Atlases have also been generated to study zebrafish (*Danio rerio*) (20) and Atlantic salmon (*Salmo salar*) (21) spleen. Additionally, scRNAseq was used to understand the effects of *A. hydrophila* infection on the innate immune system of hybrid yellow catfish (*P. fulvidraco* ♀ × *Pelteobagrus vachelli* ♂) sampled from the head kidney (17).

We set out to produce a single nuclei transcriptomic atlas of channel catfish splenic B cells to understand the underlying biology of these adaptive immune cells. Using a recombinant antibody specific for binding channel catfish IgM combined with flow cytometry, we isolated splenic IgM^+^ B cells from three individuals and paired them with a 10x Genomics microfluidics-based method of single-nuclei RNA sequencing to create an aggregated cell atlas. This atlas will provide a basis for our understanding of IgM^+^ splenic B cells specifically, and the processes that occur in these cells. Furthermore, we found that other cell types were also selected in the sorting process, likely through receptors that bind secreted IgM. Therefore, the characteristics of the cells that bind IgM on cell surface receptors are also included in this dataset.

## 2 Materials and methods

### 2.1 Animal ethics

This study was carried out at the United States Department of Agriculture – Agricultural Research Service (USDA-ARS) Aquatic Animal Health Research Unit (AAHRU). The use of fish in this study was approved by the AAHRU Institutional Animal Care and Use Committee to ensure the ethical use of research animals. The protocol conformed to USDA-ARS Policies and Procedures 130.4 and 635.1.

### 2.2 Tissue preparation

The individual channel catfish were initially processed as previously described (19). Briefly, three channel catfish (~1 kg) were euthanized by placing them in a solution of buffered MS-222 for 10 min (300 mg/L, Syndel USA, Ferndale, WA). The spleens were collected and passed through a 70 µM and 40 µM cell sieve (ThermoFisher Scientific, Waltham, MA), washed three times by suspension in Leibovitz’s L-15 medium adjusted to catfish tonicity (cL-15), and centrifuged, and the supernatant was aspirated. The cells were counted, and their viability was assessed. The cell suspensions were then layered over Lymphoprep (Serumwerk BAG, Oslo, Norway) and centrifuged at 1,200 RPM for 20 min with no brake. The buffy coat interface was aspirated, and cells were washed twice in cL-15 and centrifuged at 1,200 RPM for 5 min.

### 2.3 Flow cytometry

The isolated cells were sorted using the methods described by (22). Briefly, for single color flow cytometry, the splenic cell suspensions (1-3 × 10^7^) were incubated with 0.3 μg/mL of recombinant 9E1 monoclonal antibody (mAb) and then incubated with goat anti-mouse IgG-FITC (1:200, Southern Biotechnology, Birmingham, AL). Both antibodies were diluted with FACS buffer (1x PBS, 0.5% BSA, 2 mM EDTA) and the incubation periods were 30 min on ice. The cells were washed with FACS buffer after incubation with antibodies. Cells were resuspended in 1-2 mL of FACS Buffer and 1 μL of propidium iodide and sorted using a CytoFLEX SRT cell sorter (Beckman Coulter, Brea, CA) using lymphocyte, singlet, viability, and GFP+ and FITC gates into a tube with Leibovitz’s L-15 medium adjusted to catfish tonicity. Approximately, ~1-3 x 10^6^ sorted cells were pelleted and suspended in 1 mL of freezing medium (90% FBS, 10% DMSO). The samples were stored in cryogenic vials (Greiner Bio-One, Monroe, NC) within Mr. Frosty freezing containers (ThermoFisher Scientific, Waltham, MA) at -80°C for 24 h and then transferred to liquid nitrogen storage until they were shipped to the sequencing vendor.

### 2.4 Single-nuclei RNAseq library construction

The following steps were carried out by the sequencing vendor and described in more detail by (19). Frozen cells were thawed in a water bath set to 28°C and added to 6 mL of cL-15. The cells were centrifuged at 1,200 RPM for 5 min. The supernatant was removed, and the cells were resuspended with 1 mL of cl-15. Approximately 2.5 x 10^6^ cells were transferred to new tubes, centrifuged at 1,200 RPM for 5 min at 4°C, and the supernatant was removed. The cells were lysed in 200 µL of lysis buffer and incubated for 1 min on ice. The lysis buffer consisted of Tris-HCL at pH 7.4, with NaCl and MgCl_2_. The lysed cells were washed with 800 µL of buffer solution and centrifuged. The buffer contained BSA Solution, RNase Inhibitor, and 1x PBS. The nuclei were washed two times using 1 mL of buffer solution, pelleted, and the supernatant was removed. Centrifugation was carried out at 1,200 RPM for 10 min at 4°C. The nuclei concentration was diluted to the target concentration required for library preparation with the 10x Genomics Next-GEM Single Cell 3’ protocol (10x Genomics, Pleasanton, CA).

The single-nuclei RNA-seq libraries were prepared using the Chromium X Instrument (10x Genomics) and the Chromium Next GEM Single Cell 3’ GEM Kit v3.1 (10x Genomics) following the manufacturer’s protocol. The nuclei were added to the master mix and loaded into the Chromium Next GEM Chip G, along with the barcoded Single Cell 3’ v3.1 Gel Beads and partitioning oil. The chip was loaded into the Chromium X Instrument to generate gel beads-in-emulsion (GEMs). The gel beads were dissolved to release the primers and incubated to produce barcoded full-length cDNA. The leftover reagents were removed from the cDNA using Dynabeads MyOne SILANE (ThermoFisher Scientific, Waltham, MA) and then the cDNA was amplified via PCR. The optimal sized cDNA amplicons were selected using SPRIselect (Beckman Coulter, Brea, CA). cDNA was fragmented and Illumina indexes and adapters were added via end repair, A-tailing, adaptor ligation, and then amplified via PCR. The sample quality was assessed using a TapeStation (Agilent, Santa Clara, CA) with the TapeStation High Sensitivity D1000 ScreenTape (Agilent, Santa Clara, CA). cDNA libraries were multiplexed and sequenced using the Illumina NovaSeq X Plus sequencer (San Diego, CA).

### 2.5 Data processing

The raw data was demultiplexed to FASTQ files using ‘cellranger mkfastq’ in Cell Ranger (v5.0.0). The channel catfish genome was downloaded from the NCBI (Coco_2.0, GCF_001660625.3). The GTF annotation file was manually curated to include immunoglobulin heavy constant mu (*ighm*), as previously described (19). The annotation for *ighm* spans chr2:17,077,000-17,085,528 based on the channel catfish genomic DNA sequence of the IgM heavy chain (NCBI Accession number X52617.1) and includes four exons encoding Cμ1-4 and two exons encoding the transmembrane (TM) domains (19, 20). The genome was converted to a Cell Ranger compatible format using ‘cellranger mkref’ (Cell Ranger v8.0.0). The sequences were then trimmed, aligned to the reference assembly, filtered, and counted to generate feature-barcode matrices using ‘cellranger count’ and an argument was set to exclude introns.

A second GTF annotation file was manually curated to count membrane-bound *ighm* transcripts and secreted *ighm* transcripts separately. The membrane *ighm* annotation only included the transmembrane exons (TM1 and TM2), while the secreted *ighm* annotation only included the Cμ4 domain. These regions are unique to the respective transcripts.

Further filtering was conducted using Seurat (v5.0.1) in R (v4.3.3). First, the barcodes predicted to be duplets and multiplets were identified using the ‘scDblFinder’ function in the scDblFinder package (v1.16.0) and these barcodes were removed from the Seurat object. The barcodes that had the number of detected features outside of 3.5 median absolute deviations from the median were then removed. Barcodes that had expression from mitochondrial genes > 8% were also removed from the data as they were likely to be dying cells. The data were normalized and regularized using the ‘SCTransform’ function (sctransform, v0.4.1) and the ‘vars.to.regress’ argument was used to reduce the contribution of the percentage of mitochondrial DNA in the principal component analysis (PCA). PCA was conducted using ‘RunPCA’ and the number of dimensions used for the cluster analysis was based on the elbow plot.

### 2.6 Cluster analysis

The samples were integrated using Seurat. First, the most variable features were identified using ‘SelectIntegrationFeatures’ and then the data were prepared for integration using ‘PrepSCTIntegration’. Anchors, which are paired cells present in each dataset, were identified using ‘FindIntegrationAnchors’ and the anchors were used to perform integration with ‘IntegrateData’. The cluster analysis used the PCA dimensions 1:24 and the resolution was set to 0.5. The top 20 differentially expressed (DE) genes for each cluster against all other cells in the analysis were found using the ‘FindAllMarkers’ function and the Wilcoxon rank sum test. The P-value was adjusted for the false-discovery rate (FDR) and genes were considered DE when the adjusted P-value was < 0.05 and had a log2 fold-change (log2FC) of > 0.8. The list was further filtered to identify genes that were expressed in at least 50% of the cells in a given cluster.

### 2.7 Pathway analysis

An over-representation analysis (ORA) was conducted using clusterProfiler (version 4.12.6) for the Kyoto Encyclopedia of Genes and Genomes (KEGG) and Gene Ontology (GO) pathways (23). The ORA, which uses the hypergeometric test, was carried out using the `compareCluster` function, using the cluster as the group. The analysis included both significantly increased and decreased genes (FDR-adjusted P < 0.05, log2FC > 0.8), present in at least 20% of cells in the cluster if upregulated, or outside the cluster if the gene was downregulated.

### 2.8 Trajectory analysis

Trajectory analyses were conducted using two approaches: 1) Monocle 3 (v1.3.7) ^1^ and 2) scVelo (v0.2.5). For analysis with Monocle 3, the Seurat object with the active assay set to ‘SCT’ was converted to a cell_data_set object using the ‘as.cell_data_set’ function in the package SeuratWrappers (v0.3.5). The cell trajectories were predicted using the ‘learn_graph’ function, and the projections were plotted onto the UMAP generated from Seurat. The pseudotime was predicted using the ‘order_cells’ function. Genes that changed as a function of pseudotime were identified using the ‘graph_test’ function which utilizes Moran’s test, and significant genes had an FDR-adjusted P-value < 0.05.

The analysis with scVelo was conducted in a Python environment (v3.12.1). The Cell Ranger output and the GTF annotation for the reference assembly were used to obtain counts for spliced and unspliced mRNA for each gene using velocyto (v0.17.17). The resulting loom files containing mRNA splicing information for individuals were aggregated and combined with the cluster and UMAP information generated using Seurat. Cell velocity was inferred using scVelo using the dynamical algorithm. The cell velocities and latency were projected onto UMAP graphs generated in Seurat.

### 2.9 IgM receptor candidate gene analysis

Potential candidate genes included Fc receptor-like genes and polymeric immunoglobulin receptor genes with any level of expression in one or more of the four clusters (natural killer-like (NK-like), T cells, hematopoietic stem and progenitor cells (HSPC)/megakaryocytes (MK), and myeloids). Uncharacterized loci with immunoglobulin (Ig) domains, as predicted by the Uniprot entry (24), and significantly increased expression in these clusters were also examined. After identifying the potential candidate genes, the protein sequences for all isoforms were analyzed in InterPro (25) to identify transmembrane regions and Ig domains. Signal peptide regions were predicted using SignalP (v6.0) (26). Genes without a transmembrane domain, Ig domains, or signal peptides were removed from the candidate list. The localization of the protein in the cell was predicted with DeepLoc (v2.1) (27).

## 3 Results

### 3.1 Identifying cell types

**Figure 1:**
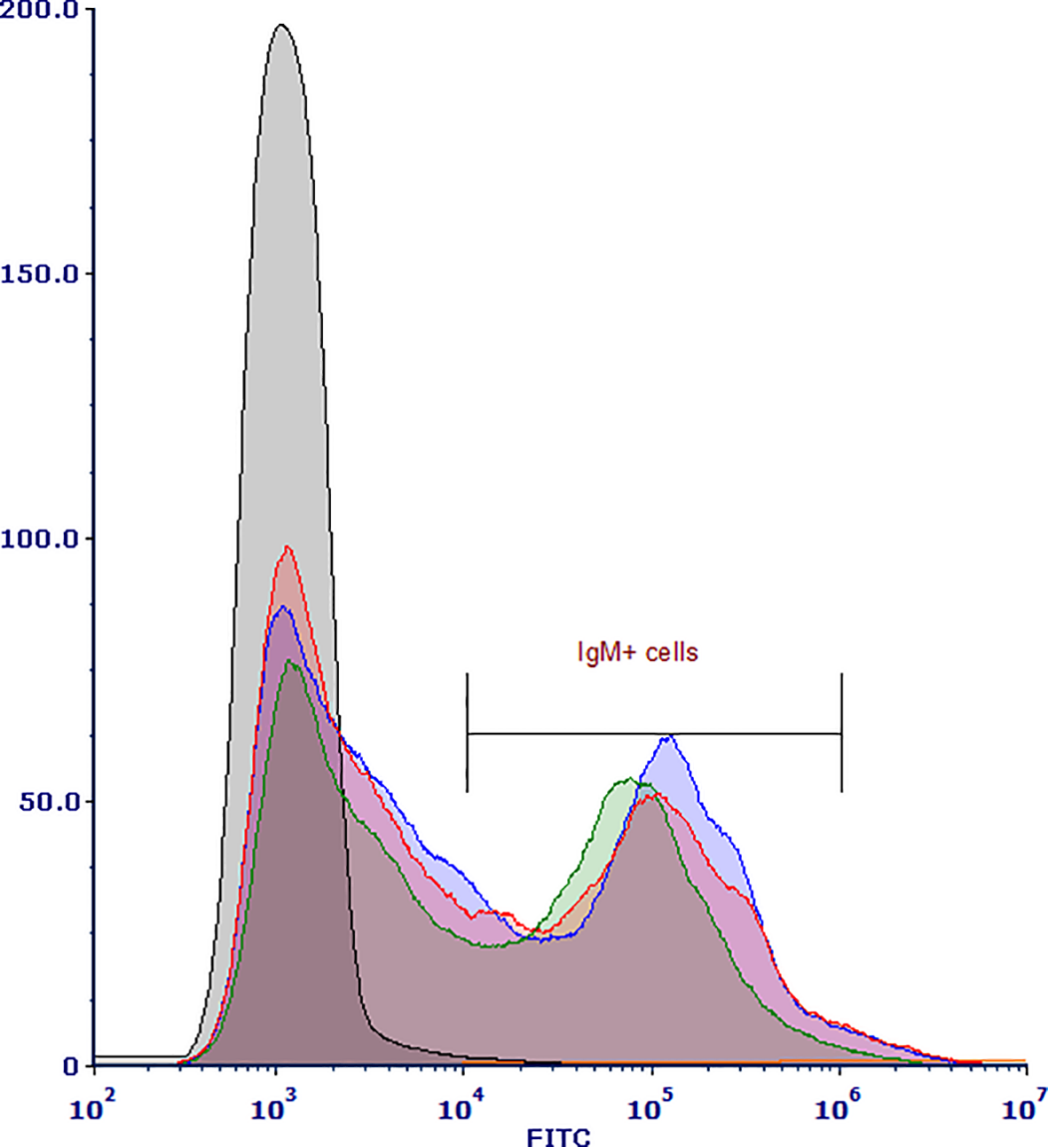
Histogram of flow cytometry-sorted channel catfish (*Ictalurus punctatus*) IgM^+^ splenic cells isolated from three healthy adults. Cell suspensions were stained with recombinant 9E1 mAb, washed, and then stained with the FITC labeled goat α-mouse IgG secondary Ab. The histogram shows the distribution of gated IgM^+^ cells in the three splenic samples (blue = sample 1, green = sample 2, red = sample 3) captured and collected by the CytoFLEX SRT.

Individual splenic lymphocyte samples stained with the r9E1 mAb recognizing catfish IgM were sorted with an average of 2.2 x 107 events processed and an average of 2.67 x 106 IgM^+^ cells sorted using the MFI range of 104–106 (**Figure 1**). Single-nuclei transcriptomes of three IgM-sorted spleen samples were produced from adult channel catfish. Across the three samples, approximately 753.5 million reads were generated for 18,686 cells. The barcode rank plots indicated that there was good separation of cell-associated barcodes and barcodes representing background noise, which were excluded from the analysis (**Supplementary Figure 1**). Overall, the sequencing and mapping parameters indicated that high quality reads were generated from the snRNAseq libraries (**Table 1**).

**Table 1:** Summary statistics of single-nuclei transcriptome libraries generated from three catfish spleens.

Sample	Output	Sample 1	Sample 2	Sample 3	Seurat aggregated samples
Number of reads	Cell Ranger	192,987,707	285,755,434	274,750,037	
Mean number of reads/cell	Cell Ranger	27,335	58,449	40,782	
Q30 bases in RNA reads	Cell Ranger	94.7%	94.7%	94.5%	
Confident alignment to reference genome	Cell Ranger	78.1%	74.1%	77.5%	
Confident alignment to reference transcriptome	Cell Ranger	53.9%	48.1%	55.2%	
Estimated number of cells	Cell Ranger	7,060	4,889	6,737	
	Seurat	4,237	2,751	3,649	10,637
Number of features detected	Cell Ranger	20,130	19,915	20,290	
	Seurat	17,016	16,735	17,225	18,632
Median number of features/cell	Cell Ranger	816	824	844	
	Seurat	795	829	801	805

The data were initially filtered to remove barcodes not associated with cells using Cell Ranger (10x Genomics) and further filtering was carried out using Seurat to remove multiplets and cells with > 8% reads aligned to mitochondrial genes. The individual libraries were integrated for combined cluster analysis in Seurat.

**Figure 2:**
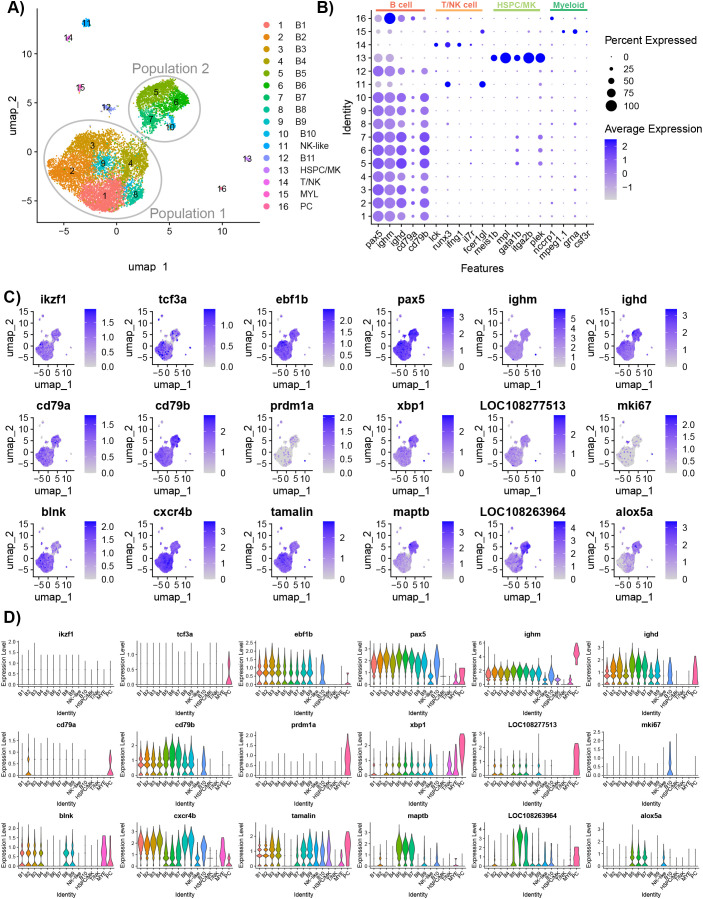
Characterization of IgM^+^ sorted spleen cells isolated from healthy adult *Ictalurus punctatus*. **(A)** UMAP plot of cells labeled by cluster. The majority of B cells are grouped into two groups: Population 1 (six clusters) and Population 2 (four clusters). **(B)** Dot plot of selected cell type markers to classify the clusters. Beside B cells, other cell types were selected by the anti-IgM and these cells may have cell surface receptors that bind secreted IgM. Dot size represents the percentage of cells expressing a gene, while color represents the average expression (z-score). **(C)** Feature plots of selected gene markers for different B cell developmental stages and genes differentially expressed between Population 1 and Population 2. Differentially expressed genes were identified by comparing cells within a cluster to all other cells using the Wilcoxon rank sum test and P-values adjusted for FDR (P<0.05). Cells expressing a given gene are visualized over non-expressing cells. **(D)** Violin plots of cluster gene expression level of markers for different B cell developmental stages and genes differentially expressed between Population 1 and Population 2.

After filtering out multiplets and cells with a high expression of mtDNA, 10,637 cells remained for the cluster analysis (**Table 1**; **Supplementary Figure 2**). The individual samples were integrated, and the cluster analysis partitioned the cells into 16 clusters (**Figure 2A**). The individual samples each contributed cells to each cluster (**Supplementary Figure 3**). The heatmap of the top 10 most upregulated genes showed that the clusters were distinct (**Supplementary Figure 4**). The cluster analysis identified B cells (10,276), NK-like cells (178), T or natural killer cells (45), HSPC/MK (66), myeloid or myeloid-derived cells (40), and plasma cells (32) (**Figure 2B**). Clusters B1–B11 and PC were identified as B cell lineage cells and were defined by their expression of paired box 5 (*pax5*), *ighm*, immunoglobulin heavy constant delta (*ighd*), CD79a molecule, immunoglobulin-associated alpha (*cd79a*), and CD79b molecule, immunoglobulin-associated beta (*cd79b*) (**Figure 2B**) (6, 10).

### 3.2 Identifying B cell developmental stages

Most of the B cells presented on the UMAP plot were in two distinct groups, defined as Population 1 and Population 2 (**Figure 2A**). These groups did not include B cell clusters B11 and PC which were positioned separately on the UMAP. To determine whether these groups represented different B cell developmental stages, canonical stage markers Ikaros (*ikzf1*), transcription factor 3 (*tcf3a*, *tcf3b*), EBF transcription factor 1 (e*bf1a*, *ebf1b*)*, pax5, ighm, ighd*, *cd79a, cd79b*, PR/SET domain 1 (*prdm1a*, *prdm1b*), x-box binding protein 1 (*xpb1*), and secreted IgD (*LOC108277513*) were examined (6, 10). There was a minimal expression of *ikzf1* and *tcf3a*/*b* across B cell clusters, except for cluster 15 which expressed *tcf3a*. Population 1 had greater expression of *ebf1b* than other B cell clusters. B3 had the greatest expression of *ikzf1* and e*bf1b*, indicating these cells likely represent the earliest B cell developmental stage in the dataset. Population 2 had a moderate expression of *xpb1* and the highest expression of *ighd* and *cd79b.* Cluster PC had the highest expression of *tcf3a*, *ighm*, *prdm1a*, *xpb1, cd79a*, and secreted IgD, and thus, these cells were predicted to be plasma cells. Paralogs *tcf3b*, *ebf1a*, and *prdm1b* had a low expression in all B cell clusters, except for the plasma cells which had the highest expression of *ebf1a*. Therefore, these genes may not have defined roles in catfish B cell development.

We further assessed the transcriptomic differences between Population 1 and Population 2 by clustering the cells with a lower resolution of 0.1. At this resolution, two populations represented one cluster each and DE genes were identified between the two clusters (**Supplementary Figure 5**; **Supplementary Table 1**). The top five DE genes in Population 1 B cells are B cell linker (*blnk*), chemokine (C-X-C motif), receptor 4b (*cxcr4b*), trafficking regulator and scaffold protein tamalin (*tamalin*), chemokine (C-C motif) receptor 9a (*ccr9a*), and phosphodiesterase 4B, cAMP-specific a (*pde4ba*). Three of these genes have known functions in cell migration (28, 29), whereas, in humans, *blnk* regulates the differentiation of pre-B cells and light chain rearrangements (30). The top five DE genes in Population 2 B cells are microtubule-associated protein tau b (*maptb*), *LOC108263964*, arachidonate 5-lipoxygenase a (*alox5a*), transgelin 2 (*tagln2*), and thymosin beta 1 (*tmsb1*). Furthermore, Population 2 had significantly increased expression of *ighd*, *cd79b*, *xbp1*, and CD40 molecule, TNF receptor superfamily member 5 (*cd40*). To determine whether the cell cycle stage contributed to the B cell clusters, the expression levels of the cell cycle genes for G2-M phases and S phase embedded in Seurat were visualized (**Supplementary Figure 6**). B11 had an above average expression of both G2-M genes (*hmgb2a*, *cdk1*, *top2a*, *cks1b*, *mki67*, *tmpob*, *anln*, *lbr*, *cenpe*, and *ctcf*) and S phase genes [*fen1*, *mcm4*, *atad2* (*LOC108256384*), *nasp*, and *rrm2* (*LOC100304963*)]. In contrast, all other B cell clusters had low expression of cell cycle genes and, except for *hmgb2a*, *mef2cb*, and *pcna*, few cells expressed these genes. Therefore, B11 likely represents a cycling B cell population.

**Figure 3:**
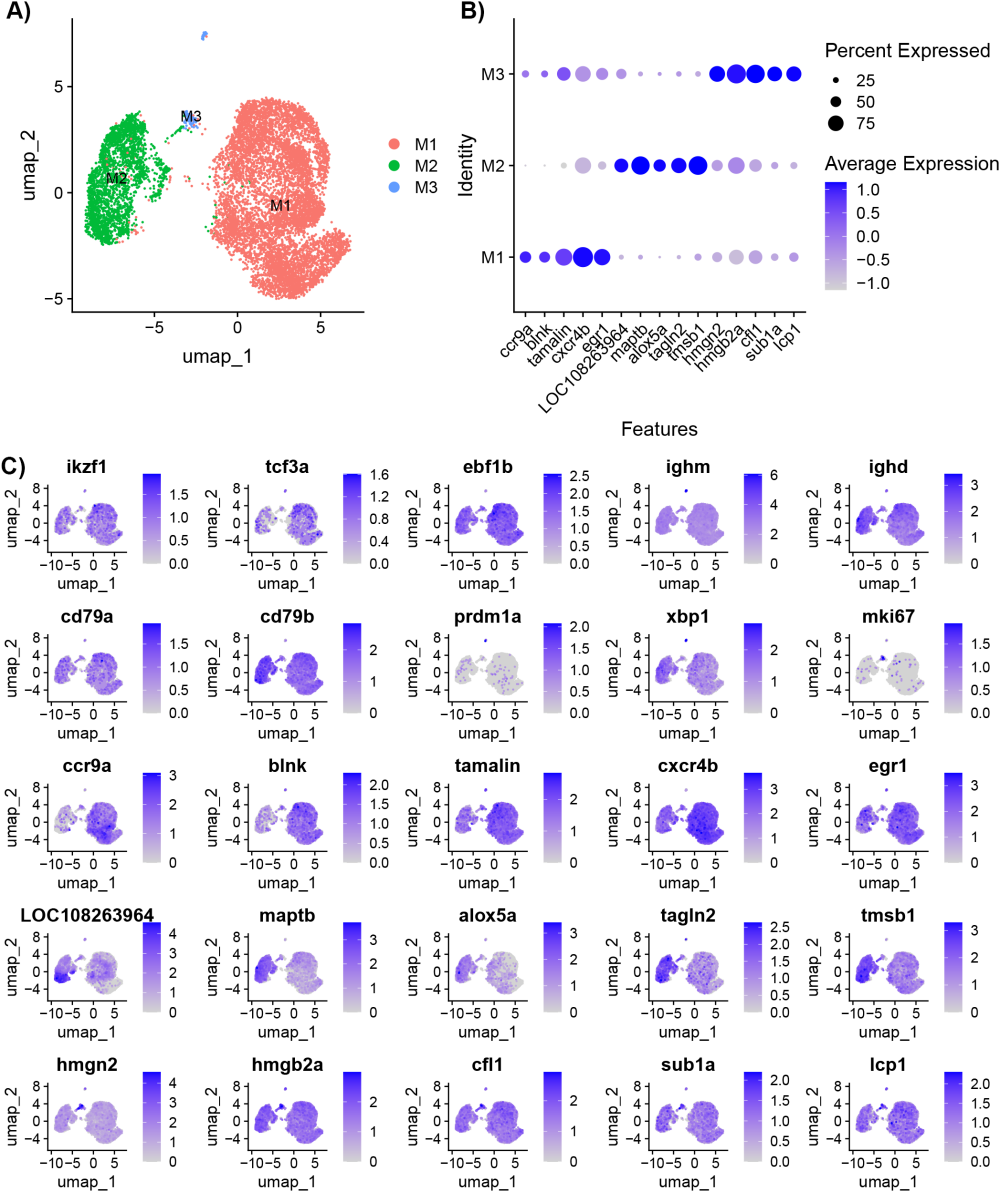
Characterization of IgM^+^ B cells isolated from healthy adult *Ictalurus punctatus*. **(A)** UMAP plot of B cells labeled by cluster. **(B)** Dot plot of the top significant highly expressed genes. Dot size represents the percentage of cells expressing a gene, while color represents the average expression (z-score). **(C)** Feature plots of selected gene markers for different B cell developmental stages and top significant highly expressed genes. Differentially expressed genes were identified by comparing cells within a cluster to all other cells using the Wilcoxon rank sum test and P-values adjusted for FDR (P<0.05). Cells expressing a given gene are visualized over non-expressing cells.

The cluster and differential gene expression analysis was repeated with only B cells to account for any noise the other cell types may contribute to the data (**Supplementary Table 2**). A resolution of 0.1 was used to separate the B cell populations into three sub-types (M1, M2, and M3) (**Figure 3A**). The M3 subset included the cycling B cells and plasma cells. The top five DE genes for M1 are *ccr9a*, *blnk, tamalin cxcr4b*, and early growth response 1 (*egr1*) (**Figures 3B, C**). *Egr1* is a zinc finger transcription factor expressed in response to BCR stimulation (31). The top five DE genes for M2 are *LOC108263964*, *maptb*, *alox5a*, *tagln2*, and *tmsb1* (**Figures 3B, C**). The top five DE genes for M3 are high mobility group nucleosomal binding domain 2 (*hmgn2*), high mobility group box 2a (*hmgb2a*), cofilin (*cfl1*), SUB1 regulator of transcription a (*sub1a*), and L-plastin (*lcp1*) (**Figures 3B, C**). High motility group (HMG) genes are involved in DNA processes such as transcription, replication, recombination, and repair (32). *Sub1a* (also known as *PC4*) has been shown to be involved in clonal expansion and differentiation of mature B cells to plasma cells (33).

**Figure 4:**
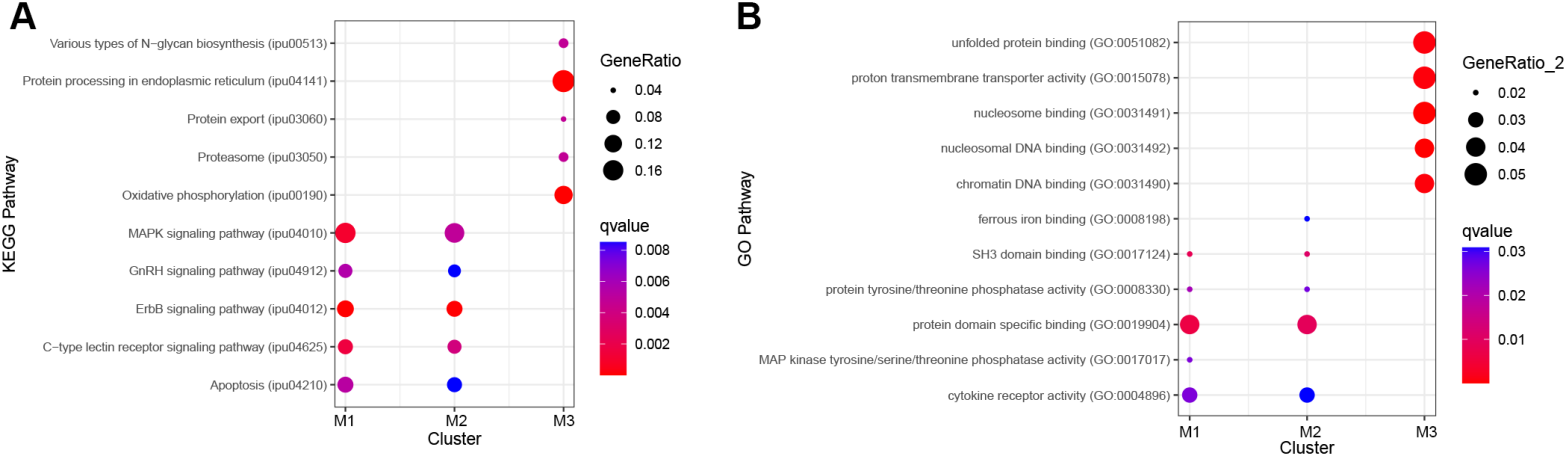
Top five significantly enriched KEGG and GO pathways for B cell clusters M1–3 using differentially expressed (DE) genes between clusters. Genes with an adjusted P-value < 0.05 and log2fc > ± 0.8 were included in the analysis. Over-representation analysis (ORA) for the **(A)** KEGG and **(B)** GO pathways used the hypergeometric test, and P-values were adjusted for FDR (P<0.05).

Pathway analysis was conducted to identify enriched pathways for the B cell clusters (M1–3). For clusters M1, M2, and M3, ORA pathway analysis identified 14, 14, and 9 enriched KEGG pathways (**Supplementary Table 3**) and 8, 7, and 19 enriched GO pathways, respectively (**Supplementary Table 4**). The top five KEGG pathways for M1 and M2 were ErbB signaling pathway, mitogen-activated protein kinase (MAPK) signaling pathway, C-type lectin receptor signaling pathway, apoptosis, and gonadotropin-releasing hormone (GnRH) signaling pathway (**Figure 4A**). For M3, the top KEGG pathways were endoplasmic reticulum, oxidative phosphorylation, various types of N-glycan biosynthesis, proteasome, and protein export (**Figure 4A**). M1 and M2 shared four of the five top GO pathways (**Figure 4B**). The shared pathways were protein domain specific binding, SH3 domain binding, protein tyrosine/threonine phosphatase activity, and cytokine receptor activity. The unique pathway for M1 was MAP kinase tyrosine/serine/threonine phosphatase activity while the unique pathway for M2 was ferrous iron binding. The top five GO pathways for M3 were nucleosomal DNA binding, nucleosome binding, chromatin DNA binding, proton transmembrane transporter activity, and unfolded protein binding (**Figure 4B**).

### 3.3 B cell classes

Given that the mAb 9E1 selects for IgM^+^ cells, IgM^+^ B cells and IgM^+^IgD^+^ B cells are expected within the data set. However, the B cells did not appear to cluster by expression of *ighm* or *ighd*, though, a portion of cells appeared to express both loci, while some cells only expressed *ighm*.

**Figure 5:**
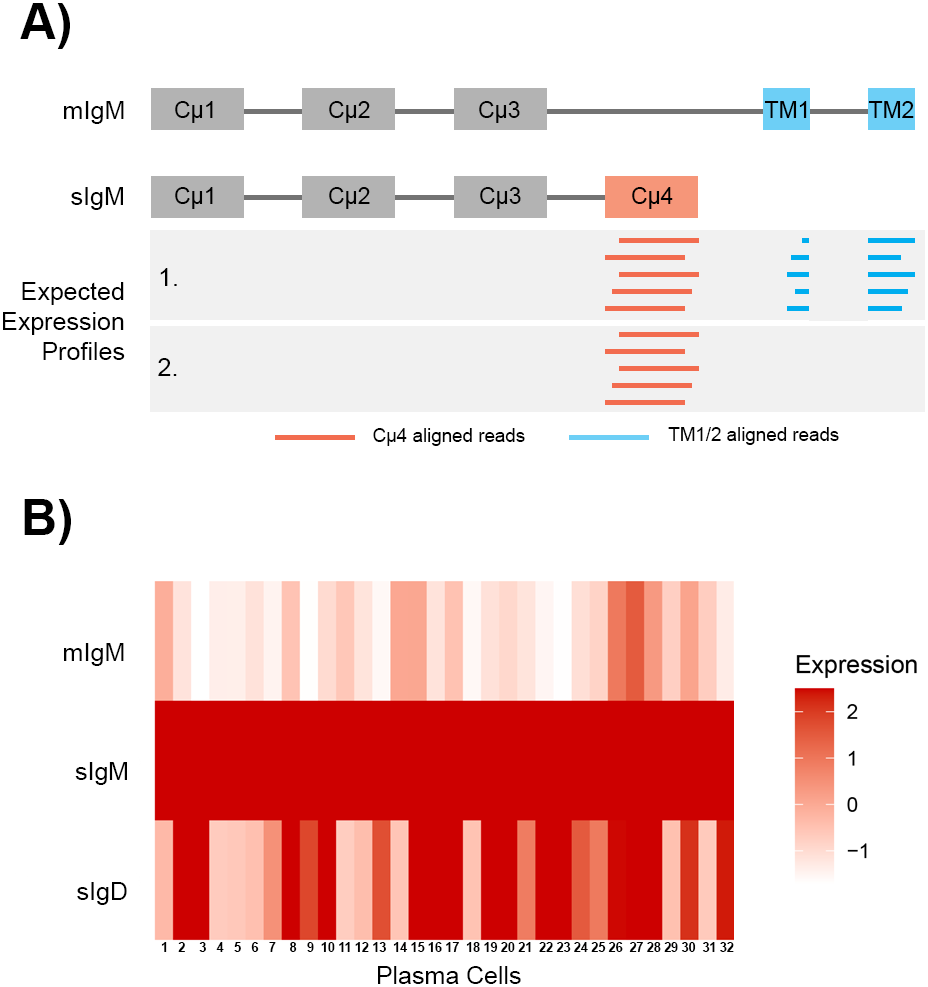
Expression of membrane IgM (mIgM), secreted IgM (sIgM), and secreted IgD (sIgD) in 32 plasma cells. **(A)** Schematic of the exonic structures of mIgM and secreted IgM. The aligned reads are depicted as horizontal lines in the grey box. Reads that aligned to the transmembrane exons (TM1 and TM2) were counted for the mIgM (blue), while reads that aligned to the fourth constant domain (Cμ4) were counted for the sIgM (red). IgM (chr2:17,077,000-17,085,528bp) was annotated based on the GenBank accession X52617.1. The exon annotations spanned chr2:17,080,455-17,080,843 for Cμ4, chr2:17,082,738-17,082,872 for TM1 and chr2:17,085,220-17,085,258 for TM2. **(B)** The 32 plasma cells highly expressed sIgM and had low expression of mIgM. Some plasma cells also highly expressed sIgD. Expression is presented as z-scores calculated from all cells in the atlas.

The plasma cells (cluster PC) have the highest expression of *ighm* and the increased expression represented the upregulation of secreted *ighm* transcript. To differentiate between the membrane and secreted transcripts, the *ighm* annotation in the channel catfish Coco_2.0 GTF file was modified so that reads aligning to the transmembrane exons (TM1 and TM2) were counted for membrane *ighm* and reads that aligned to the Cµ4 domain were counted for secreted *ighm* (**Figure 5A**), as the *ighm* membrane transcript does not include the Cµ4 domain. The reads aligning to Cµ1-3 were not counted as they are common between the membrane and secreted *ighm* transcripts. For the plasma cells in cluster 15, the expression of secreted *ighm* was highly expressed compared to membrane *ighm* (**Figure 5B**), confirming that there was a switch in expression from membrane to secreted. Unlike IgM, the secreted form of IgD is expressed by another locus, *LOC108277513* (also known as *IGHD3*), and was also significantly expressed by plasma cells (**Figures 2C**, **5B**). In contrast, the expression of *ighd* was significantly reduced (adj P-value = 0.00, log2fc = -2.62) in the plasma cells.

### 3.4 Transcriptional and pathway characterization of B cell clusters

The DE genes among the 16 clusters were assessed to further characterize the B cell clusters (**Supplementary Table 5**). Expression was compared between the cluster of interest and all other cells. The DE genes discussed were filtered using strict parameters to identify key genes for each cluster (adjusted P-value < 0.05, log2fc > ± 0.8, and percentage of cells expressing gene > 50%). For KEGG and GO pathway enrichment, a more relaxed criterion was used (percentage of cells expressing gene > 20%) to include lowly expressed genes.

B1 only had four DE genes: eukaryotic translation elongation factor 1 beta 2 (*eef1b2*), ribosomal protein S17 (*rps17*), chemokine (C-C motif) receptor 9a (*ccr9a*), and partner of NOB1 homolog (*pno1*) were significantly upregulated in B1. *Eef1b2* and *pno1* are involved in translation (24, 34), while *ccr9* is a chemokine receptor involved in migration (28). There were no significant pathways in the ORA for the DE genes.

**Figure 6:**
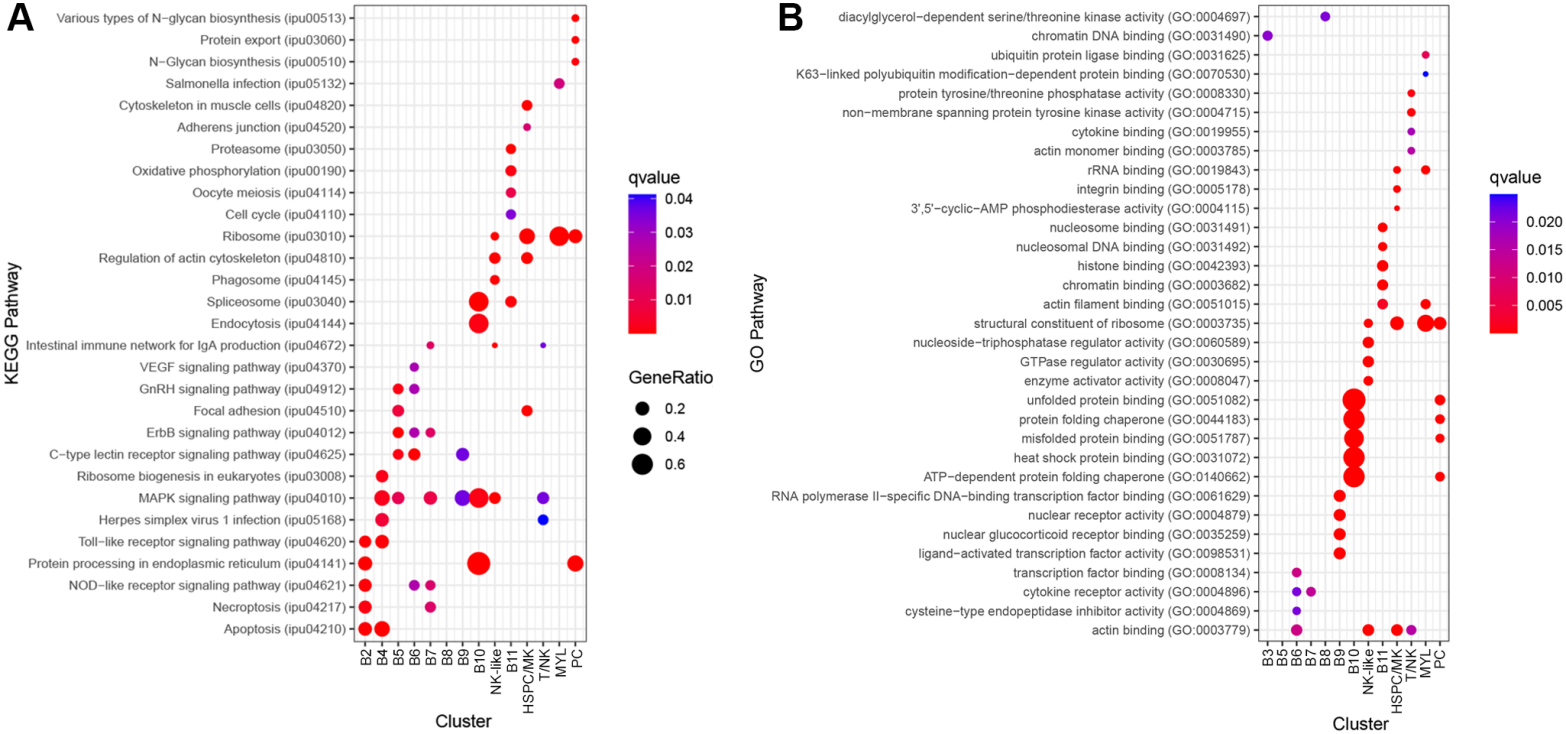
Top 5 significantly enriched KEGG and GO pathways for each cluster, using differentially expressed (DE) genes between clusters. Genes with an FDR-adjusted P-value < 0.05 and log2fc > ± 0.8 were included in the analysis. Over-representation analysis (ORA) for the **(A)** KEGG and **(B)** GO pathways used the hypergeometric test, and P-values were adjusted for FDR (P<0.05).

There were three genes with B cell functions among the top DE genes for B2. These were actin, beta 2 (*actb2*), early growth response 2b (*egr2b*), and signal sequence receptor, delta (*ssr4*) (35–37). Two other DE genes were associated with translation: eukaryotic translation initiation factor 3 subunit E, a (*eif3ea*) and eukaryotic translation initiation factor 3, and subunit D (*eif3d*) (38). ORA pathway analysis identified 12 enriched KEGG pathways for B2 (**Supplementary Table 6**). The top five pathways were protein processing in the endoplasmic reticulum, apoptosis, NOD-like receptor signaling pathway, toll-like receptor signaling pathway, and necroptosis (**Figure 6A**).

B3 significantly expressed B cell-related genes, ring finger and CCCH-type domains 1a (*rc3h1a*; aka Roquin), *pde4ba*, insulin receptor substrate 2 (*irs2b*), inositol polyphosphate-4-phosphatase type I Ab (*inpp4ab*), A-kinase anchoring protein 13 (*akap13*), and nuclear receptor subfamily 4, group A, member 3 (*nr4a3*). The functions of these genes include differentiation, survival, and suppression of B cell activation (39–45). ORA pathway analysis identified one enriched GO pathway for B3, chromatin DNA binding (**Figure 6B**; **Supplementary Table 7**).

B4 highly expressed C-C motif chemokine 3 (*LOC108268454*; also known as *CD193*), CD83 molecule (*cd83*), B cell lymphoma 2 like 10 (*bcl2l10*), lysine (K)-specific demethylase 6B, a (*kdm6ba*; also known as *JMJD3*), nuclear factor of kappa B inhibitor, alpha (IκBα) paralogs (*nfkbiaa* and *nfkbiab*), nuclear factor kappa B subunit 2 (*nfkb2*; NFκB2), and *nr4a3*. These mostly have functions in B cell maturation and activation (46–51). Furthermore, in the spleen, *cd83*, *nfkbiaa*, and *nfkbiab* are involved in marginal zone (MZ) B cell maturation (46, 51). ORA pathway analysis identified six enriched KEGG pathways for B4 (**Supplementary Table 6**). The top five pathways were apoptosis, toll-like receptor signaling pathway, ribosome biogenesis in eukaryotes, MAPK signaling pathway, and herpes simplex virus 1 infection (**Figure 6A**).

Population 2 cell clusters B5, B6, and B7 had common DE genes. In addition to the genes mentioned in section 3.2 (*alox5a*, *maptb*, *tmsb1*, *tagln2*), *LOC108271063* [adhesion G-protein coupled receptor G5 (*adrg5*)], *itm2b*, *LOC100528069* (ferritin middle subunit), *glyr1*, and *vaspa* were upregulated in these clusters. These genes do not have known functions in B cells. B5 and B6 had two common DE genes, kruppel-like factor 2 (*klf2a*) and tyrosine 3-monooxygenase/tryptophan 5-monooxygenase activation protein, theta polypeptide a (*ywhaqa*). *Klf2a* is first upregulated in pre-B cells and suppresses B cell activation (52). B5 and B7 had three common differentially expressed genes, ADP-ribosylation factor-like 5C (*arl5c*), RAB11 family interacting protein 1 (class I) b (*rab11fip1b*), and ER degradation enhancer, mannosidase alpha-like 3 (*edem3*). *Rab11fip1* may play a role in B cell proliferation and apoptosis inhibition (53).

B5 had significantly greater expression of three additional genes with B cell functions, *ighd*, phosphoinositide-3-kinase, regulatory subunit 1 (*pik3r1*), and basic helix-loop-helix family, member e40 (*bhlhe40*). *Pik3r1* and *bhlhe40* are involved in B cell differentiation (54, 55), and *bhlhe40* specifically functions as a negative regulator of germinal center B cells, a type of B cell not wholly observed in teleosts. ORA pathway analysis identified seven enriched KEGG pathways for B5 (**Supplementary Table 6**). The top five pathways were ErbB signaling pathway, C-type lectin receptor signaling pathway, GnRH signaling pathway, focal adhesion, and MAPK signaling pathway (**Figure 6A**).

B6 has significantly higher expression of components of the BCR complex, *cd79b* and immunoglobulin lambda-1 light chain (*LOC108260251*) (56). The anti-apoptotic factor in B cells, BCL2 apoptosis regulator a (*bcl2a*), is also significantly upregulated (57). ORA pathway analysis identified eight enriched KEGG pathways (**Supplementary Table 6**) and four enriched GO pathways for B6 (**Supplementary Table 7**). The top five KEGG pathways were the C-type lectin receptor signaling pathway, ErbB signaling pathway, NOD-like receptor signaling pathway, VEGF signaling pathway, and GnRH signaling pathway (**Figure 6A**). The GO pathways were transcription factor binding, actin binding, cytokine receptor activity, and cysteine-type endopeptidase inhibitor activity (**Figure 6B**).

B7 was characterized by increased expression of C-C motif chemokine 3 (*LOC108268454*; also known as *CD193*), syndecan 4 (*sdc4*), nuclear receptor subfamily 4, group A, member 1 (*nr4a1*), *cd40*, MYC proto-oncogene, bHLH transcription factor (*mycb*), solute carrier family 1 member 5 (*slc1a5*; also known as *ASCT2*). These genes include cell surface receptors, and have functions in apoptosis, survival, differentiation and maturation of B cells (44, 58–61). ORA pathway analysis identified nine enriched KEGG pathways (**Supplementary Table 6**) and one enriched GO pathway for B7 (**Supplementary Table 7**). The top five KEGG pathways were MAPK signaling pathway, intestinal immune network for IgA production, ErbB signaling pathway, necroptosis and NOD-like receptor signaling pathway (**Figure 6A**). The significant GO pathway was cytokine receptor activity (**Figure 6B**).

B8 had five significantly increased genes and only two had known B cell functions, Src like adaptor 2b (*sla2b*; also known as *SLAP2*) and *ccr9a*. The role of *sla2b* function in B cells is not known; however, it appears to function downstream of the BCR complex (62). ORA pathway analysis identified one enriched GO pathway for B8 (**Supplementary Table 7**), diacylglycerol-dependent serine/threonine kinase activity (**Figure 6B**).

B9 has significantly increased expression of nuclear receptor subfamily 4, group A, member 1 (*nr4a1*, *LOC108255274*), *nr4a3*, RELB proto-oncogene, NFκB subunit (*relb*), fosB proto-oncogene (*fosb*), *egr2b*, *tamalin*, Src like adaptor (*LOC108270945*), LYN proto-oncogene, Src family tyrosine kinase (*lyn*), lysine (K)-specific demethylase 6B, a (*kdm6ba*), and dual adaptor of phosphotyrosine and 3-phosphoinositides (*dapp1*). These genes have roles in regulating immune tolerance, survival, migration, B cell development, and response to antigen stimulation (44, 62–67). In mammals, *kdm6ba* and *dapp1* are expressed by germinal center B cells (66, 67). ORA pathway analysis identified two enriched KEGG pathways (**Supplementary Table 6**) and four enriched GO pathways for B9 (**Supplementary Table 7**). The KEGG pathways were the MAPK signaling pathway and C-type lectin receptor signaling pathway (**Figure 6A**). The GO pathways were nuclear glucocorticoid receptor binding, RNA polymerase II-specific DNA-binding transcription factor binding, nuclear receptor activity, and ligand-activated transcription factor activity (**Figure 6B**).

B10 was defined by increased expression of four heat shock proteins: heat shock 70 kDa (*LOC108259092*, *LOC128629166;* also known as HSP70), DnaJ heat shock protein family member B1b (*dnajb1b*; also known as HSP40) and heat shock protein 90, alpha (*hsp90aa1.2*; also known as HSP90). HSP70, HSP40, and HSP90 are chaperone molecules and play a role in protein folding (68–70). In humans, HSP70 is highly expressed in regulatory B cells and secreted to suppress effecter cells (71). ORA pathway analysis identified four enriched KEGG (**Supplementary Table 6**) and 11 GO pathways for B10 (**Supplementary Table 7**). The KEGG pathways were protein processing in the endoplasmic reticulum, spliceosome, endocytosis, and MAPK signaling pathway (**Figure 6A**). The top five pathways were unfolded protein binding, ATP-dependent protein folding chaperone, protein folding chaperone, heat shock protein binding, and misfolded protein binding (**Figure 6B**).

B11 were identified as cycling B cells and the top DE genes included four high motility group (HMG) genes: *hmgn2*, *hmgb2a*, non-histone chromosomal protein HMG-like (*si:ch73-1a9.3*) and high mobility group nucleosomal binding domain 7 (*hmgn7*). These genes are involved in DNA processes such as transcription, replication, recombination, and repair (32). Additionally, structural proteins were DE which supports the identity of these cells as cycling B cells. These proteins include prothymosin alpha (*si:ch211-222l21.1*), *cfl1*, coactosin-like F-actin binding protein 1 (*cotl1*), *lcp1*, actin, beta 1 (*actb1*), prothymosin alpha b (*ptmab*), coronin, actin binding protein, 1A (*coro1a*), and tubulin, beta 2b (*tubb2b*). ORA pathway analysis identified five enriched KEGG pathways (**Supplementary Table 6**) and seven enriched GO pathways for B11 (**Supplementary Table 7**). The top five KEGG pathways were proteasome, spliceosome, oxidative phosphorylation, oocyte meiosis, and cell cycle (**Figure 6A**). The top five GO pathways were histone binding, nucleosomal DNA binding, nucleosome binding, chromatin binding, and actin filament binding (**Figure 6B**).

Cluster PC highly expressed B stage marker genes *prdm1a*, *xbp1*, and *ighm*. Other highly expressed genes included selenoprotein M (*selenom*), protein disulfide isomerase family A, member 4 (*pdia4*), protein disulfide isomerase family A, member 3 (*pdia3*) peroxiredoxin 4 (*prdx4*), and dual specificity phosphatase 5 (*dusp5*). *Selenom, pdia4*, and *pdia3* are highly expressed by human plasma cells (36). *Prdx4* may be involved with light chain synthesis and *dusp5* may have a key role in the differentiation of B cells to plasma cells (72, 73). ORA pathway analysis identified eight enriched KEGG pathways (**Supplementary Table 6**) and 16 GO pathways for Cluster 15 (**Supplementary Table 7**). The top five KEGG pathways were protein processing in the endoplasmic reticulum, ribosome, protein export, various types of N-glycan biosynthesis, and N-Glycan biosynthesis (**Figure 6A**). The top five GO pathways were protein processing in structural constituent of ribosome, unfolded protein binding, ATP-dependent protein folding chaperone, misfolded protein binding, and protein folding chaperone (**Figure 6B**). B10 and PC have seven common enriched GO pathways: ATP hydrolysis activity, ATP-dependent protein folding chaperone, heat shock protein binding, misfolded protein binding, protein folding chaperone, ubiquitin protein ligase binding, and unfolded protein binding. Therefore, B10 may represent B cells beginning differentiation into plasma cells.

### 3.5 MAPK, GnRH, and NOD-like receptor pathway genes were differentially regulated

To investigate the signaling pathways in B cells, we selected some enriched pathways of interest and evaluated the expression pattern of genes across the clusters. These pathways were MAPK, GnRH and NOD-like receptor (NLR) signaling.

The MAPK cascade is a highly conserved pathway involved in cell functions including proliferation, differentiation, migration, and apoptosis. MAPK signaling was significantly enriched for Population 1 B cell clusters B4 and B9, and Population 2 B cell clusters B5–B7 and B10 (**Supplementary Table 6**). DE genes of interest within this pathway included *dusp2*, *dusp6*, *nfkb2, maptb, jun*, and *june.* Dusp2 and dusp6 are negative regulators of the MAPK signaling. *Dusp2* has significantly increased expression in B4 and decreased in B5 and B6, while *dusp6* has significantly decreased in B5–B7. *Nfkb2*, *jun*, and *june*, genes encoding transcription factors that promote proliferation and inflammation and protect the cell against apoptosis, are also differentially expressed. *Nfkb2* encodes a component of the NFκB and is upregulated in B4 and B7. *Jun* is significantly downregulated in B5-B7 and *june* has significantly increased expression in B5. MAPK signaling was also significantly enriched for clusters M1 and M2 generated from the sub-cluster analysis of B cells (**Figure 3**). Consistent with the analysis for all cells, *dusp2*, *dusp6*, and *jun* were among the highly expressed genes in this pathway for cluster M1 (**Supplementary Table 3**). In addition, the gene encoding PKCβ (*prkcbb*) was highly expressed for M1 and while *nfkb2* was highly expressed for M2. In B cell activation, PKCβ results in the nuclearization of NFκB (74). Therefore, NFκB may be retained in the cytoplasm unless nuclearized by another signaling pathway. Further, M2 cells also highly expressed *dusp1*, *mapkapk3*, and *rap1b*. Like dusp2/6, dusp1 also negatively regulates MAPK signaling. In mammals, rap1b has several B cell functions, including homeostasis of MZ B cells, migration and T-dependent humoral response. Taken together, MAPK signaling may be occurring in both Population 1/M1 and Population 2/M2 B cells but gene expression demonstrates differential regulation of the pathway.

GnRH signaling includes mechanisms that overlap with BCR signaling, including diacylglycerol (DAG) activation of protein kinase C, beta (PKCβ), and Ca^2+^ influx regulation (74). The pathway also includes MAPK signaling. GnRH signaling is only enriched in the Population 2 B cell B5-7, and the key gene encoding PKCβ (*prkcbb*), is significantly downregulated in these cells. In these clusters, calcium/calmodulin-dependent protein kinase (CaM kinase) II delta 1 (*camk2d1*) has significantly greater expression, suggesting that this gene is involved in the calcium signaling in Population 2 B cells. GnRH signaling was also significantly enriched for clusters M1 and M2 generated from the sub-cluster analysis of B cells (**Figure 3**). Consistent with the analysis for all cells, *prkcbb* and *camk2d1* contribute to the enrichment of this pathway. Additionally, transcription factors enriched in this pathway are differentially expressed. *Egr1* and *jun* are significantly expressed in M1 cells, while *june* is significantly expressed in M2 cells. Therefore, key genes in this pathway are differentially expressed between the Population 1/M1 and Population 2/M2 B cells.

NLR signaling was significantly enriched for B1, B6 and B7. Differently regulated genes in this pathway include *bcl2a*, baculoviral IAP repeat containing 2 (*birc2;* also known as cIAP), TNF receptor-associated factor 2b (*traf2b*) which have higher expression in the B cells B6-B7. In the pathway, BCL2A negatively regulates the inflammasome NLRP1, though a channel catfish ortholog for NLRP1 has not been identified. In mammals, NOD1/2 recruits RIP2 to promote inflammatory responses (75). In this process, RIP2 undergoes ubiquitination which may be regulated by TRAF2 and cIAP (75). A deubiquitinating enzyme called TNFAIP3 (encoded by *tnfaip3*; also known as A20) may also regulate RIP2 (75). *Tnfaip3* is significantly downregulated in B2. Therefore, catfish rip2 ubiquitination, as a part of the NLR signaling pathway, may be differentially regulated in the B cells.

### 3.6 Trajectory of B cells

The analysis of the B cells suggested that there were mature B cells to plasma cells. The trajectory analysis was conducted using two approaches: 1) ordering cells based on gene expression changes (Monocle 3) and 2) ordering cells based on changes in ratios of unspliced and spliced RNA (scVelo).

**Figure 7:**
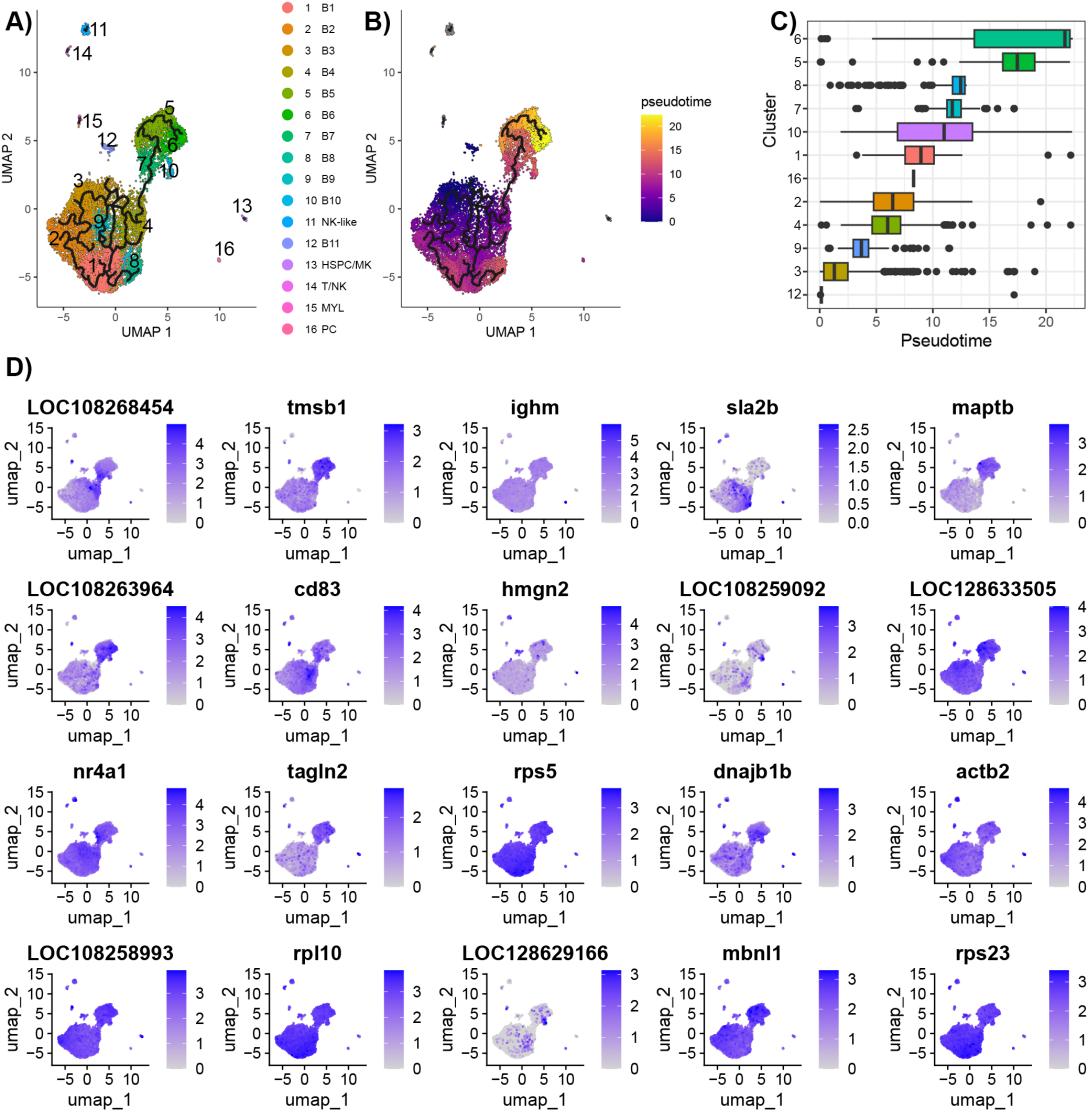
Trajectory analysis of B cells predicted using Monocle 3. **(A)** Branching between clusters visualized on the UMAP generated in Seurat. B cells were in the same partition, and other cell types (NK-like, HSC/MK, T/NK, and MYL) each had separate partitions. **(B)** Pseudotime ranking of individual cells. Nodes in B3 were selected as the root as this cluster had the highest expression of *ebf1b*, a marker of immature B cells. The ranking agrees with the expression of B cell development markers. Pseudotime was only calculated for B cells. **(C)** Box plots of pseudotime for each cluster. **(D)** Feature plot of genes found to significantly change expression as a function of pseudotime using Moran’s test.

The branching predicted by Monocle 3 showed that there are dynamic cell processes occurring for the B cells (**Figure 7A**). The Population 1 and Population 2 B cells are connected via B4 and B7. Pseudotime analysis ranks the cells based on cell processes, and in this case, represents differentiation. As cluster B2 had the greatest expression of *ikzf1* and *ebf1b*, the nodes in this cluster were selected at the root cells. Other cell types (NK-like, HSC/MK, T/NK, and MYL) were not included in the pseudotime calculations. The pseudotime demonstrated that the cell trajectory flowed from Population 1 to Population 2, with an endpoint in cluster B6 as indicated by the greatest pseudotime (**Figure 7**). Genes with significant expression changes as a function of pseudotime included C-C motif chemokine 3 (*LOC108268454*), *ighm*, *maptb*, *cd83*, *hmgn2*, and *cd74b* (**Figure 7D**; **Supplementary Table 8**).

To explore further the trajectory of the B cells, the analysis with Monocle 3 was repeated on the subset of B cells and plasma cells (**Figure 3**; **Supplementary Figure 7**; **Supplementary Table 9**). The branch in cluster M1 with the greatest expression of *ikzf1* and *ebf1b* was chosen as the root. The analysis demonstrated branching points within M1, and a branching point that connects the three populations. In addition, the pseudotime analysis suggests that there are end points in all three clusters.

The analysis conducted using scVelo on all cells (**Figure 2A**) showed that the cell velocity flowed from the Population 1 clusters, beginning from B1, towards the mature clusters and end point in B5 (**Supplementary Figure 8A**). The pseudotime was consistent with the velocity map, showing that B1 had the smallest pseudotime and B5 had the greatest pseudotime. Consistent with the Monocle 3 analysis, there was no significant connection between the B cells and the plasma cells. The scVelo dynamical model is programmed to handle datasets with populations with diverse cell types (76).

### 3.7 Antibody r9E1 selects other cell types in the spleen

Cells in clusters labeled NK-like, HSPC/MK, T/NK, and MYL do not express B cell markers (**Figures 2B–D**; **Supplementary Table 5**). Therefore, the antibody, mA r9E1, used for sorting cells selected for some additional cell types. These cells may have been picked up by the secreted IgM bound to a surface receptor, such as a membrane Fcµ receptor (FcmR) or polymeric immunoglobulin receptor (pIgR). A gene encoding membrane FcmR has not yet identified in channel catfish; however, a soluble form has been characterized (77).

The NK-like cells significantly expressed *fcer1gl*, therefore, this population is predicted to be natural killer-like cells (78). Furthermore, the cluster highly expressed genes typical of natural killer cells such as NK-lysin type 1 (*LOC100304648*), killer cell lectin-like receptor subfamily E member 1 (*LOC108266645*), perforin 1 (*LOC108280700*), filamin A, alpha (*flna*), and FYN binding protein b (*fybb*) (79–82). ORA pathway analysis identified 20 enriched KEGG pathways (**Supplementary Table 6**) and 23 GO pathways (**Supplementary Table 7**) for the NK-like cluster. The top five KEGG pathways were the regulation of actin cytoskeleton, intestinal immune network for IgA production, MAPK signaling pathway, ribosome, and phagosome (**Figure 6A**). The top five GO pathways were actin binding, GTPase regulator activity, nucleoside-triphosphatase regulator activity, enzyme activator activity, and structural constituent of ribosome (**Figure 6B**).

**Figure 8:**
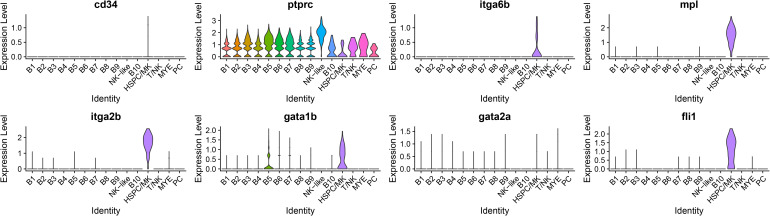
Violin plots of genes that are expressed by populations of HSPC. The cluster of HSPC/MK expressed genes corresponding to both hematopoietic stem and progenitor cells (HSPC) and megakaryocytes (MK). In an attempt to classify the cells, HSPC marker expression, including *cd34*, *ptprc* (cd45ra), and *itga6b* (cd49f), was assessed. Further, markers for MK biased HSPC were assessed and three genes, *itga2b*, *gata1b*, and *fli1*, were expressed by the cluster. *Mpl* encodes a receptor that regulates megakaryocytopoiesis from HSPCs.

The HSPC/MK cluster was initially classified as potentially HSPC due to the expression of meis homeobox 1 (*meis1b*), MPL proto-oncogene, thrombopoietin receptor (*mpl*), GATA binding protein 1 (*gata1b*), integrin, alpha 2b (*itga2b*), and pleckstrin (*plek*). In addition, other markers for various HSPC populations were expressed (**Figure 8**), including *cd45ra* (*ptprc*), *cd49f* (*itga6b*), and a low expression of *cd34* and *cd71* (*tfr1b*) (83, 84). The DE genes for this cluster are expressed by HSPC and (MK). Highly expressed genes include *mpl*, glycoprotein Ib platelet subunit beta (*gp1bb*), thrombospondin 1b (*thbs1b*), apelin (*apln*), *itga2b*, platelet glycoprotein Ib alpha chain (*LOC108275690*), myosin, light chain 9b, regulatory (*myl9b*), coagulation factor II (thrombin) receptor (*f2r*), and endoglin (*eng*) (85–93). Markers of MK biased HSPC include *itga2b*, *gata1*, GATA binding protein 2 (*gata2b*), von Willebrand factor (*vwf*), KIT proto-oncogene, receptor tyrosine kinase (*kita/kitb*), growth factor independent 1B transcription repressor (*gfi1b*), and fli-1 proto-oncogene, ETS transcription factor (*fli1*) (94). Only *itga2b*, *gata1b*, and *fli1* were detected (**Figure 8**). Combining the maker expression and DE genes analysis, the identity of these cells remains unclear and could either represent HSPC or MK cells. ORA pathway analysis identified seven enriched KEGG pathways (**Supplementary Table 6**) and eight GO pathways (**Supplementary Table 7**) for the HSPC/MK cluster. The top five KEGG pathways were ribosome, regulation of actin cytoskeleton, focal adhesion, cytoskeleton in muscle cells, and adherens junction (**Figure 6A**). The top five GO pathways were structural constituent of ribosome, actin binding, rRNA binding, integrin binding, and 3’,5’-cyclic-AMP phosphodiesterase activity (**Figure 6B**).

The cells in the T/NK have gene expression consistent with T cells. The cells express T/NK cell markers LCK proto-oncogene, Src family tyrosine kinase (*lck*), RUNX family transcription factor 3 (*runx3*), and interferon gamma 1 (*ifng1*). Two components of the T cell receptor complex, T-cell surface glycoprotein CD3 delta chain (*LOC108278320*) and T-cell surface glycoprotein CD3 epsilon chain (*LOC108278371*), also have significantly greater expression. A cd3ζ-like gene (*cd247l*) is also highly expressed. Cd3ζ is primarily expressed by NK cells and T cells (95). Therefore, the *cd247l* gene may function as a component of the TCR complex, or alternatively, according to the Uniprot database, it may encode an Fc-gamma receptor (24). Approximately 50% of these cells significantly express granzyme K (*gzmk*), which suggests that these are effector T cells (96). ORA pathway analysis identified three enriched KEGG (**Supplementary Table 6**) and five enriched GO pathways (**Supplementary Table 7**) for T/NK. The KEGG pathways were intestinal immune network for IgA production, MAPK signaling pathway, and herpes simplex virus 1 infection (**Figure 6A**). The GO pathways were non-membrane spanning protein tyrosine kinase activity, protein tyrosine/threonine phosphatase activity, actin monomer binding, actin binding, and cytokine binding (**Figure 6B**).

MYL cells expressed myeloid lineage genes, macrophage expressed 1, tandem duplicate 1 (*mpeg1.1*), granulin a (*grna*), and colony stimulating factor 3 receptor (*csf3r*). Significantly expressed genes had functions in myeloid-derived cells and epithelial cells, therefore, this cluster is likely a mixture of cell types. Genes with myeloid functions included solute carrier family 40 member 1 (*slc40a1*; also known as *FPN1*), Kruppel-like factor 6a (*klf6a*), serglycin (*srgn*), CCAAT enhancer binding protein beta (*cebpb*), JunB proto-oncogene, AP-1 transcription factor subunit b (*junbb*), protein strawberry notch homolog 2 (*si:ch73-63e15.2*: also known as *SBNO2*), and BCL2 modifying factor 2 (*bmf2*) (36, 97–100). Genes with functions in epithelial cells were keratin pairs, keratin 8 (*krt8*) and keratin 18b (*krt18b*), and ictacalcin-like gene (*LOC108265566*). These keratins are typically expressed by simple epithelial cells (101). Ictacalcin, first identified in channel catfish, is part of the S100 gene family and is a calcium-binding protein (102). ORA pathway analysis identified two enriched KEGG (**Supplementary Table 6**) and 10 enriched GO pathways for the cluster MYL (**Supplementary Table 7**). The KEGG pathways were ribosome and salmonella infection (**Figure 6A**). The top five GO pathways were structural constituent of ribosome, rRNA binding, actin filament binding, ubiquitin protein ligase binding, and K63-linked polyubiquitin modification-dependent protein binding (**Figure 6B**).

### 3.8 IgM receptor candidate genes

We attempted to identify cell surface receptors that may bind IgM and allow the selection of the cells in clusters NK-like, HSPC/MK, T/NK, and MYL. In mammals, three genes from the polymeric immunoglobulin receptor (PIGR) gene family can bind IgM: Fc µ receptor (*FCMR*), Fc α and µ receptor (*FCAMR*), and polymeric immunoglobulin receptor (*PIGR*) (103).

Candidate genes were selected based on the criteria detailed in section 2.9. No candidate genes were expressed across all four clusters and had Ig binding domains and transmembrane domains. Therefore, more than one receptor is likely contributing to the selection of the non-B cell clusters. After analysis of the protein domains, 22 genes remained with Ig binding regions, a transmembrane domain, and a signal peptide (**Supplementary Table 10**). The prediction of the protein associations with membranes suggested that proteins were peripheral or had lipid anchors. However, the proteins were predicted to be most likely associated with the endoplasmic reticulum, nucleus, or mitochondria. These genes may contribute to the selection of the additional cell types in our analysis, but experimental validation is required.

## 4 Discussion

To investigate the underlying biology of splenic B cells, we produced an *I. punctatus* IgM^+^ spleen single-nuclei atlas. This is in addition to the single-nuclei atlas of three whole spleen samples extracted from adult channel catfish (19). Here, spleen samples from the same catfish were sorted for IgM^+^ cells and sequenced to a greater depth. By sorting the cells, a higher number of B cells were sequenced and analyzed. This study analyzed 10,308 B cell transcriptomes sequenced to an average depth of 42,189 reads per cell. The B cells were grouped into 11 clusters and were analyzed for DE genes and enriched GO and KEGG pathways. In comparison, the whole spleen atlas included 2,469 B cells assigned to nine clusters and the whole atlas was sequenced to an average depth of 34,584 (19).

Our analysis identified two major distinct populations of B cells (Populations 1 and 2), cycling B cells and plasma cells (**Figure 2**). Population 1 B cells highly expressed *ccr9a*, *blnk*, *tamalin*, *cxcr4b*, and *pde4ba*. The genes *ccr9a* and *cxcr4b* are chemokine receptors and may have a role in the migration of these cells to or from the spleen. For example, *ccr9* is involved in the migration of immature T cells to the thymus (104). Alternatively, *ccr9* may also induce migration to the gut. *Cxcr4* may induce migration to the central nervous system (29). Some murine B cells undergoing V(D)J arrangements isolated from bone marrow expressed *cxcr4* (105). A sub-cluster analysis of the B cells also showed that *egr1* is highly expressed by this group of B cells (presented as the M1 cluster in **Figure 3B**). Population 2 B cells highly expressed *maptb*, *LOC108263964*, *alox5a*, *tagln2*, and *tmsb1*. *Alox5a* is a regulator of mature naïve B cells maintenance (106). Demonstrated in mice and humans, *tagln2* expression in B cells may increase T cell activation (107). The B cell transgelin-2 accumulates at the junction of B cells and T cells (107). Thymosin proteins are small peptides expressed in all cells and have functions in migration of cells (108). Based on our analyses, we suspect that Population 1 represents transitional B cells and Population 2 represent mature B cells, though further investigation and validation needs to be conducted. Our analysis of B cells is consistent with our whole spleen atlas (19), where we identified two major groups of B cells. Some genes defining the populations were consistent. For example, *cxcr4b* and *ccr9a* are highly expressed in the larger B cell population (Population 1), while *maptb* and *alox5a* are highly expressed in the smaller population (Population 2).

Pathway analysis revealed differentially regulated signaling pathways for B cells such as MAPK and NLR signaling. The MAPK signaling pathway was significantly enriched in B cell clusters; however, DE genes were opposed between the two groups. For example, inhibitors of the MAPK pathway, *dusp2* and *dusp6*, had significantly decreased expression in the mature B cell clusters, while *dusp1* had increased expression. *Maptb*, a gene that is highly expressed by the mature B cells, may be phosphorylated as a result of MAPK signaling. Tau, encoded by *maptb*, does not have a known function in B cells but is expressed by neurons and lung cells and associated with Alzheimer’s disease (109). In the context of Alzheimer’s disease, some studies have found associations between B cells and tau; however, the relationship is yet to be defined (110, 111).

Plasma cells were also identified in the atlas. We demonstrated that these cell highly expressed sIgM and some cells also expressed low levels of mIgM. Therefore, we were able to validate co-expression of mIgM and sIgM at the individual cell level for channel catfish plasma cells in the spleen. In channel catfish, the expression of both IgM forms by IgM^+^ peripheral blood lymphocytes (PBLs) cells have been identified (7). The IgM^+^ PBLs were selected using the same antibody (mAb 9E1) used in the current study, showing that it selects antibody secreting cells (ASCs) (7). Our finding is consistent with teleost (112, 113) and mammalian literature (114) where IgM^+^ ASCs have been observed. Compared to the *I. punctatus* whole spleen data (19), there is a lower percentage of plasma cells detected in the IgM^+^ atlas (32 cells; 0.031%) than the whole spleen (125 cells; 0.5% of B cells). Therefore, the anti-IgM may be selecting a subset of plasma cells (113). The plasma cells highly expressed *selenom*, *pdia4*, *pdia3*, *prdx4*, and *dusp5*, and, similarly, these genes are highly expressed in human plasma cells (36). *Selenom* is an endoplasmic reticulum-resident oxidoreductase that may have functions in Ca^2+^ homeostasis and energy metabolism (115). While *pdia4* and *pdia3* are known to catalyze protein folding and cleavage of thiol-disulfide bonds in the endoplasmic reticulum. Though, one study has found that protein disulfide isomerase (Pdi) in Japanese lancelet (*Branchiostoma japonicum*) acted as an immunocompetent factor against bacterial species, *Escherichia coli* and *Staphylococcus aureus* (116). The authors demonstrated that Pdi had bacterial agglutination properties and was bactericidal towards *S. aureus* (116). In channel catfish, another gene in this family *pdia6* was found to be highly expressed in the head kidney, intestine, liver, and spleen after challenges with *Edwardsiella tarda*, *Streptococcus iniae*, and channel catfish reovirus (117). Therefore, *pdia4* and *pdia3* may have immune functions beyond the endoplasmic reticular roles in plasma cells. *Prdx4* expression is induced in B cell neoplasms and associated with the synthesis and secretion of immunoglobulin light chains (72). *Dusp5* may play a key role in the differentiation of B cells to plasma cells when there is BCR stimulation, and T cells help through IL-2 and IL-5 signaling (73). Sub-cluster analysis of B cells showed that the cycling B cells and plasma cells had similar gene expression profiles. Therefore, the cycling B cells may represent activated B cells undergoing clonal expansion.

Other cell types were also identified in the atlas and assigned to NK-like, HSPC/MK, T/NK, and MYE. Clusters HSPC/MK and MYE are tentatively labeled as the analysis of gene expression patterns was not conclusive. We explored potential reasons for these cells to be selected. First, our data shows that there was low expression of *ighm* in NK-like, HSPC/MK, and MYE. Given these cells are not B cells, it is unlikely that the cells express *ighm* (78) and this may be a technical artifact as abundant transcripts can be incorrectly barcoded to other cells. The cells could have sIgM binding to Ig receptors such as FcµR or PIGR on the cell surface. However, a bona fide membrane-bound Fc µ receptor has not been detected in channel catfish, only a soluble FcµR homolog (118). FcRs can activate and inhibit immune responses in immune cells such as macrophages and lymphocytes (118). PIGR is typically expressed by mucosal epithelial cells, not immune cells, and the lamina propria-facing receptors enable transcytosis of antibodies to the lumen (103). The PIGR genes in teleosts may be more closely related to CD300, another gene within the double-disulfide Immunoglobulin SuperFamily (103). We attempted to identify candidate receptors within our data but were unable to identify genes that were expressed across all four clusters. Therefore, if a receptor is responsible for the selection of these cells, then multiple candidates may be responsible. The selection of NK-like cells and T cells by mAb r9E1 has been reported previously (78, 119). However, HSPC, MK, or myeloid cells have not been reported thus far. B cells may also be sorted using antibodies to the F-type and G-type Ig light chains, as B cells only express one light chain isotype, while other cell types are double positive (78). However, the mAb 9E1 is readily available in our laboratory so studying this antibody is relevant for our research group.

This atlas provides an insight into the gene expression of IgM^+^ immune cells in channel catfish, which is a commercially and evolutionary relevant teleost species. The atlas is publicly available and could be used garner more important information regarding the gene expression of immune cells, such as expression patterns of specific gene families. Our interpretation of data is limited primarily to knowledge derived from mammals, namely humans and mice. While there are many consistencies between teleosts and mammals, the unique features of *I. punctatus* and teleost B cells are yet to be revealed in functional studies. Therefore, our analysis may act as a starting point for functional studies. This atlas will continue to be a valuable resource as our understanding of genes and pathways at the individual cell resolution develop.

## Data Availability

The datasets presented in this study can be found in online repositories. The names of the repository/repositories and accession number(s) can be found below: https://www.ncbi.nlm.nih.gov/, Gene Expression Omnibus (GEO), GSE278995.

## Footnotes

1
https://cole-trapnell-lab.github.io/monocle3/.
