# Supplementary material for: Single-nuclei transcriptome analysis of IgM+ cells isolated from channel catfish (Ictalurus punctatus) spleen
Source: Front Immunol. 2025 Mar 17;16:1547193. doi: 10.3389/fimmu.2025.1547193 (PMC11955638; doi:10.3389/fimmu.2025.1547193)
Supplement: Supplementary file 1 [file Table1.docx]

Supplementary Material

# Supplementary Figures and Tables

## Supplementary Figures


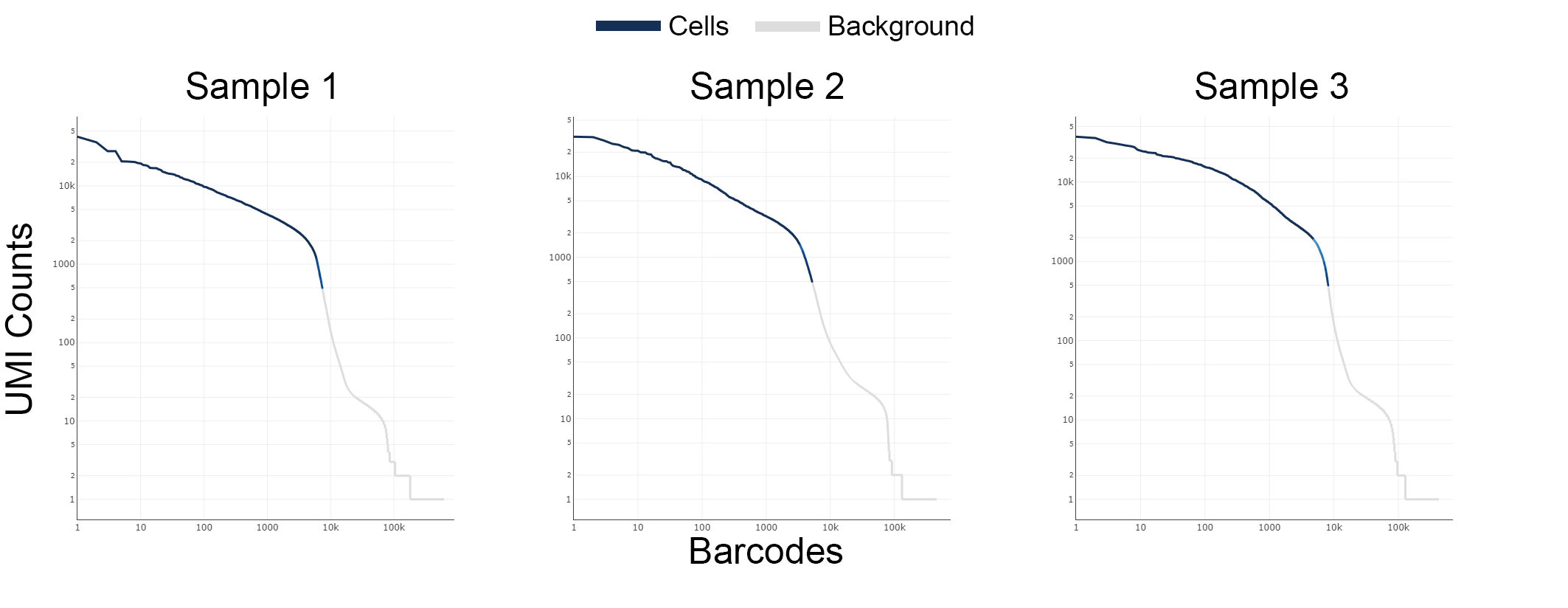
**Supplementary Figure 1: Barcode rank plot for IgM^+^ spleen cells.** The plots indicate that the sample qualities are good as there is decent separation between barcodes with high and low UMI counts, indicated by the steep decline in UMI counts as barcode rank increases.


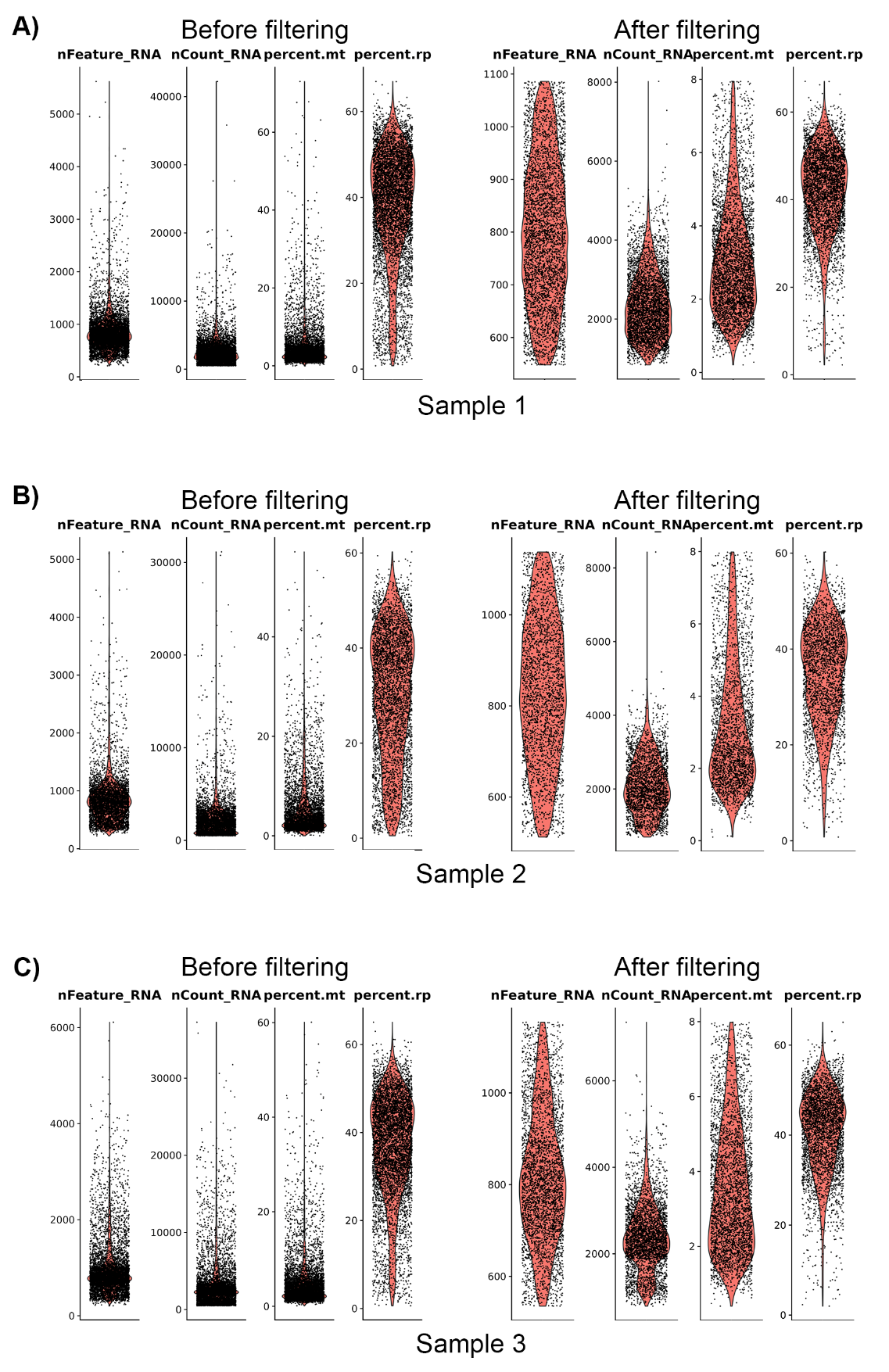


**Supplementary Figure 2:** **Sample parameters (number of features, RNA counts, percent mitochondrial genes, and percent ribosomal proteins) before and after filtering.** A) Sample 1. B) Sample 2. C) Sample 3.

**
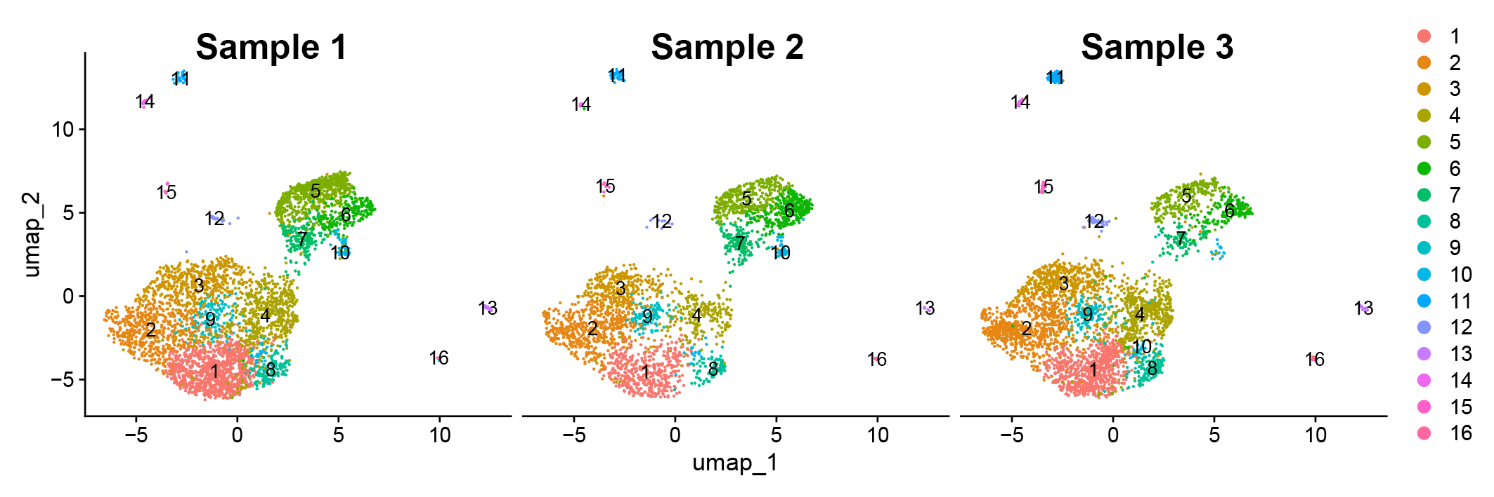
**

**Supplementary Figure 3:** **Sample contribution to the integrated dataset.** All samples contribute cells to each cluster.


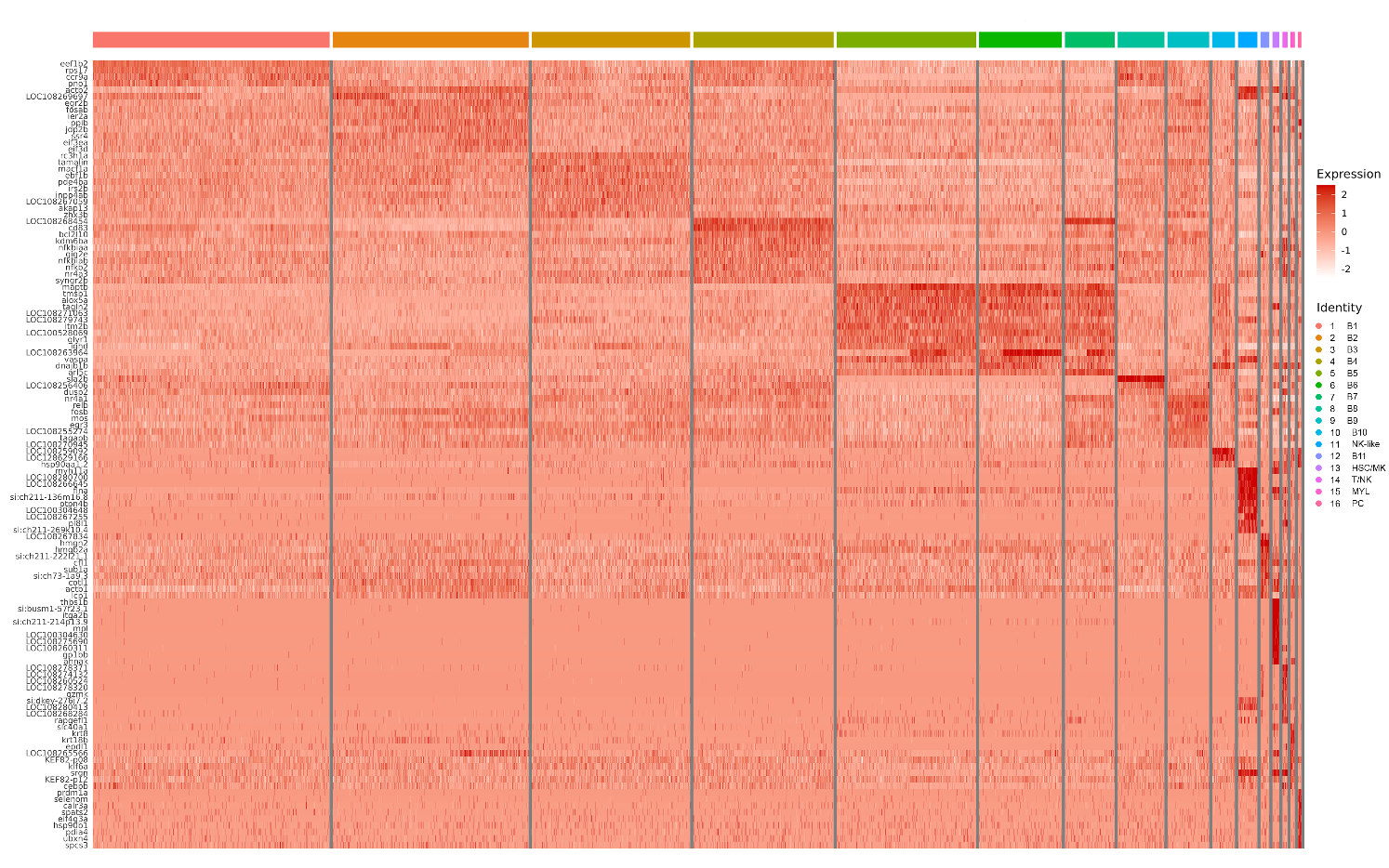


**Supplementary Figure 4.** Heatmap of top significantly upregulated genes between clusters. The heat map shows that there is good separation of genes between most clusters.


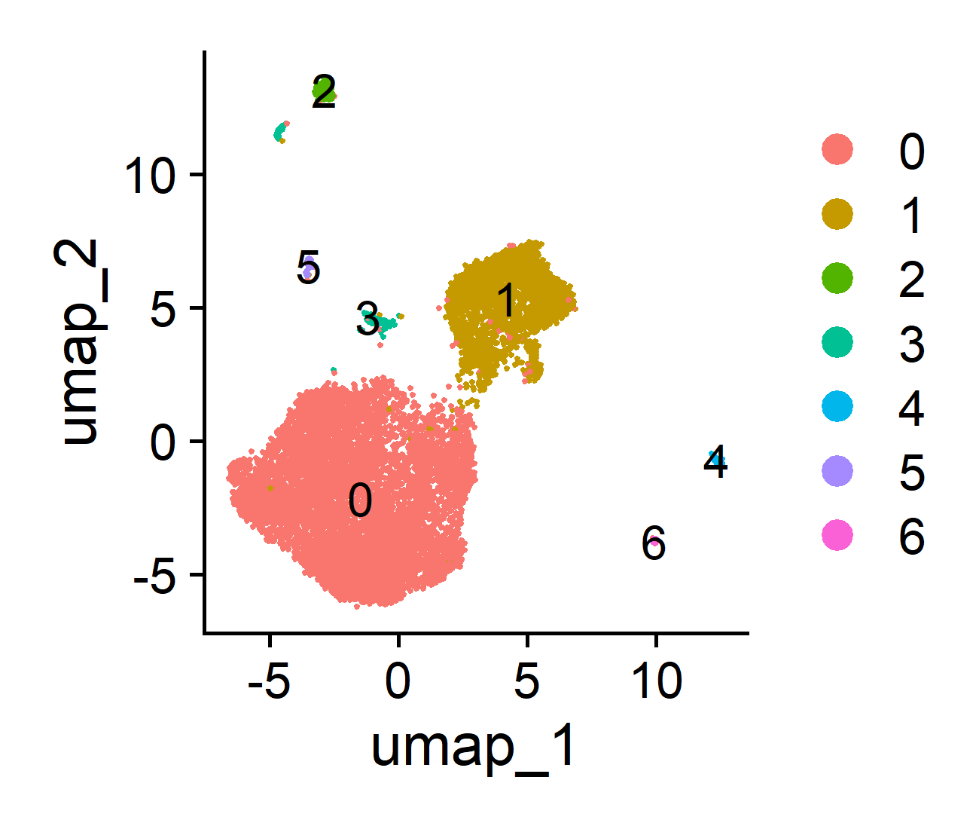


**Supplementary Figure 5:** Cluster analysis of all cells with the resolution set to 0.1. Cluster 0 represents B cells in Population 1while cluster 1 represents B cells in Population 2.


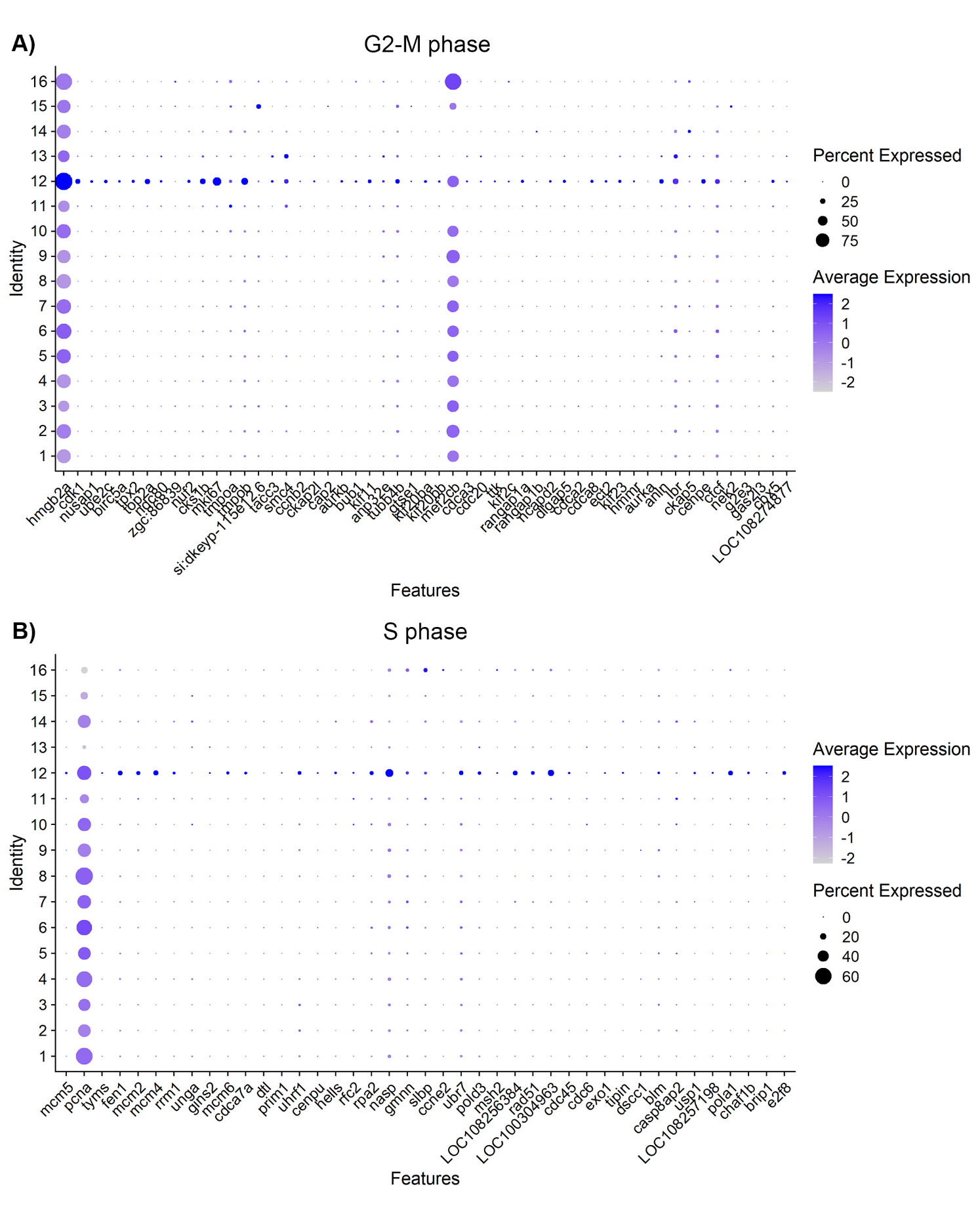


**Supplementary Figure 6: Dot plot displaying expression (z-score) of cell cycle genes across the clusters.** Cluster 11 (B cells) has greater expression of A) G2-M phase genes and B) S phase genes compared to all other clusters, suggesting that this cluster represents cycling B cells.


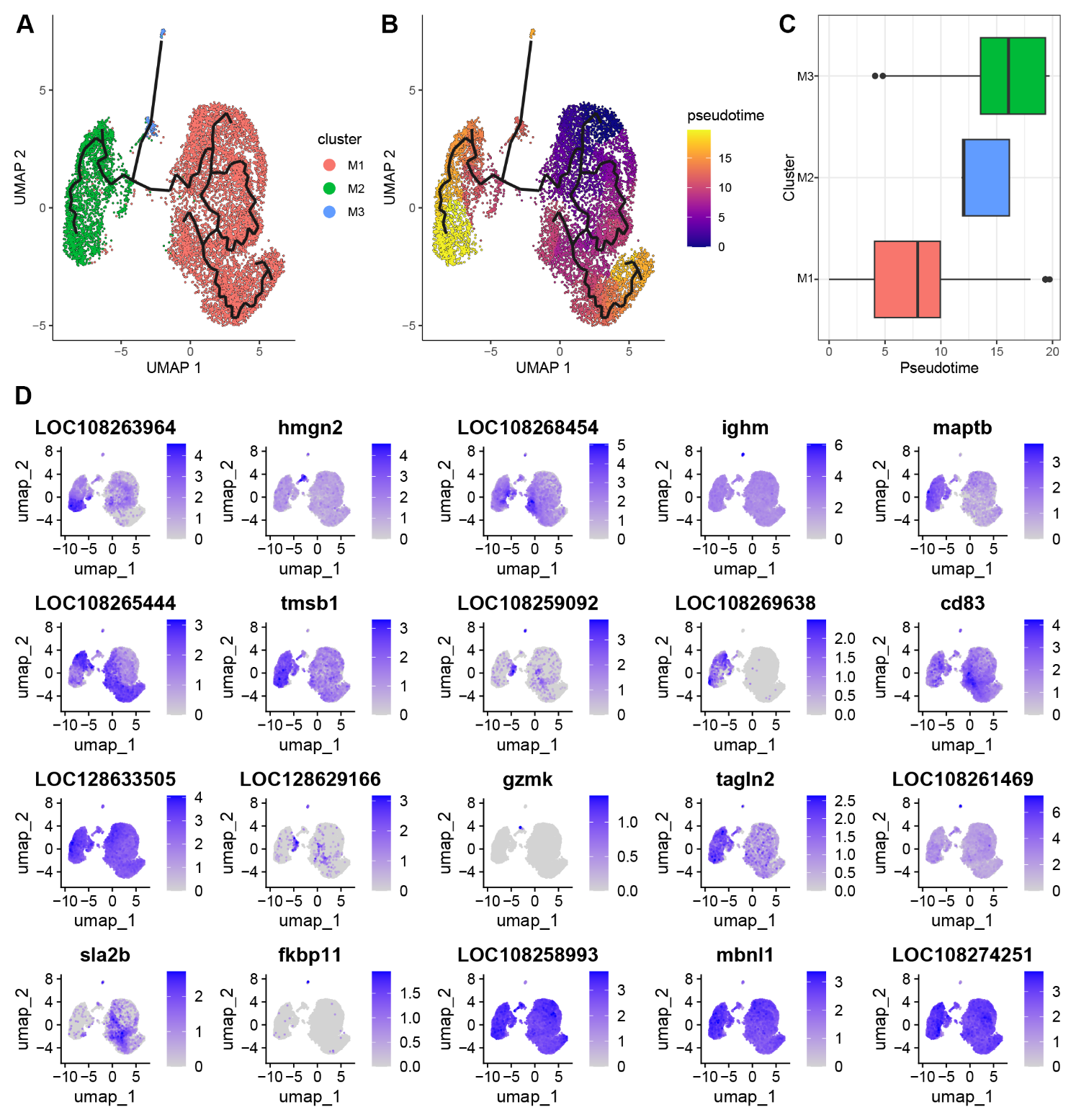


**Supplementary Figure 7: Trajectory analysis of IgM^+^ cells predicted using scVelo (A)** Velocity map displaying the direction of cell velocities. **(B)** Velocity pseudotime ranking of individual cells, where 0 indicates the earliest biological time in the dataset and 1 indicates the latest biological time. **(C)** Percentage of spliced and unspliced transcripts in total (pie chart) and by cluster (bar graph).


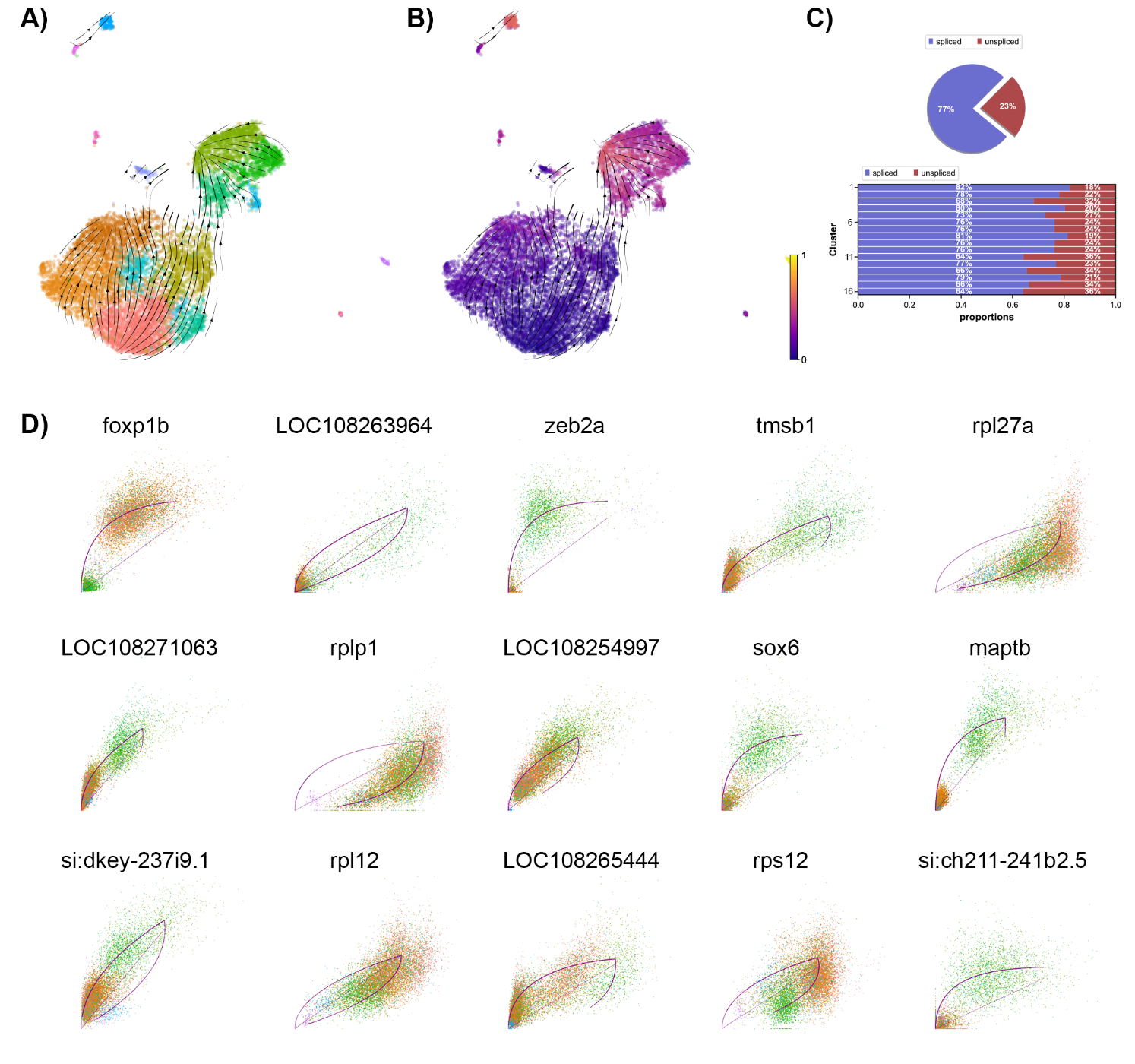


**Supplementary Figure 8: Trajectory analysis of B cells predicted using Monocle 3** (A) Branching between B cell clusters visualized on the UMAP generated in Seurat. (B) Pseudotime ranking of individual cells. Nodes in M1 with cells highly expressing ebf1b, a marker of B cells early in development, were selected as the root. (C) Box plots of pseudotime for each cluster. (D) Feature plot of genes found to significantly change expression as a function of pseudotime using Moran’s test.
